# Tongxinluo capsule for acute myocardial infarction: a systematic review and meta-analysis

**DOI:** 10.3389/fphar.2025.1632809

**Published:** 2025-10-22

**Authors:** Yang Wu, Jingxue Guo, Jiasai Fan, Xian Wang

**Affiliations:** ^1^ Department of Cardiology, Dongzhimen Hospital, Beijing University of Chinese Medicine, Beijing, China; ^2^ Institute of Cardiovascular Diseases, Beijing University of Chinese Medicine, Beijing, China

**Keywords:** Tongxinluo, acute myocardial infarction, meta-analysis, clinical studies, preclinical studies

## Abstract

**Objective:**

To evaluate the clinical and preclinical effects of Tongxinluo Capsule (TXL) on acute myocardial infarction (AMI) and to summarize reported mechanisms of action, thereby informing clinical decision-making and future research.

**Methods:**

A comprehensive computerized search of eight databases and four clinical trial registries was performed from their inception until 1 April 2025. Data extraction, quality assessment and analysis were conducted in strict accordance with predefined protocols. The methodological quality of included studies was evaluated using the RoB-2 and SYRCLE tools. Statistical analyses were carried out using RevMan 5.4 software, employing fixed-effect or random-effects models as appropriate.

**Results:**

This review included 54 clinical studies (4,353 patients in trail group; 4,296 patients in control group) and 11 animal studies (95 animals in trail group; 94 animals in control group). Meta-analysis of clinical studies indicated that, compared with control groups, TXL was associated with lower all-cause mortality, cardiovascular mortality, and incidence of myocardial reinfarction (P < 0.05). Compared with control groups, TXL was associated with lower incidence of repeated revascularization, heart failure, angina pectoris, and arrhythmias (P < 0.05). Furthermore, TXL demonstrated greater improvement in cardiac function indicators and vascular endothelial function (P < 0.001), alongside significant reductions in blood lipids levels (TC, TG, HDL-C, LDL-C; P < 0.05) and inflammation markers. TXL was associated with fewer adverse reactions (P = 0.01), primarily gastrointestinal in nature. In animal studies, TXL was correlated with lower myocardial infarction area and the no-reflow area (P < 0.001), higher cardiac function indicators (LVEF and LVFS; P < 0.05) and better vascular endothelial function (P < 0.05) compared to control group. Reported biological mechanisms of TXL include inhibition of apoptosis, suppression of inflammation, protection of cardiomyocytes and endothelial cells, promotion of angiogenesis, and synergistic lipid-lowering and plaque-stabilization effects.

**Conclusion:**

This study is the first meta-analysis to integrate both clinical and animal research on TXL for AMI. The finding suggests that TXL may be associated with alterations in left ventricular systolic function and clinical prognosis, potentially mediated through mechanisms such as inhibition of apoptosis, protecting endothelial function, reducing of inflammation, preservation of cardiomyocytes, and promotion of angiogenesis. Limitations of the included studies constrain the strength of these conclusions, and further validation through larger, high-quality randomized controlled trials is warranted.

## 1 Introduction

Acute myocardial infarction (AMI) remains a leading cause of mortality worldwide, with its incidence rising annually, thereby imposing a substantial societal burden and presenting a critical public health challenge. Ischemic heart disease, including AMI, continues to be the primary contributor to global cardiovascular mortality. Although age-standardized mortality rates have declined between 1990 and 2022, the absolute number of annual deaths increased from 12.4 million to 19.8 million during this period (Mensah et al., 2023). Reperfusion therapies such as percutaneous coronary intervention (PCI) and thrombolysis can salvage at-risk myocardium and limit infarct size in AMI. However, if myocardial ischemia is prolonged, reperfusion itself may exacerbate myocardial ischemia/reperfusion injury (MIRI). During MIRI, extensive infiltration of neutrophils and platelets occurs; and activated neutrophils release oxygen free radicals, proteolytic enzymes, and pro-inflammatory mediators, directly causing tissue and endothelial damage. This leads to lumen occlusion and microvascular dysfunction, impairing myocardial perfusion and resulting in the coronary no-reflow phenomenon (NRP). NRP occurs in approximately 36% of AMI cases, with microcirculatory dysfunction observed in up to 67% of patients (Durante et al., 2017). NRP is associated with higher rehospitalization rates, adverse ventricular remodeling, malignant arrhythmias, and heart failure, thereby diminishing the benefits of myocardial reperfusion and increasing mortality (Tasar et al., 2019). The complex pathophysiology and individual variability of MIRI and NRP pose significant challenges for developing effective preventive and treatment strategies. Further research is needed to discover multi-targeted therapeutic agents that improve endothelial function and suppress inflammatory responses, ultimately enhancing clinical outcomes in AMI.

Tongxinluo Capsule (TXL) is a Chinese patent medicine developed based on the Traditional Chinese Medicine (TCM) theory of collateral disease. Its primary functions are to invigorate qi and blood, unblock collaterals, and relieve pain. It is particularly suited for AMI, which in TCM is characterized by qi deficiency and blood stasis obstructing the collaterals. TXL is a compound capsule composed of 12 medicinal materials (Han and Zhang, 2004; Wen et al., 2004; Xiao et al., 2002). When used as an adjunct to guideline-directed medical therapy (GDMT) for AMI, TXL has been investigated for its potential to protect cardiac function and improve patient prognosis (7–60). Potential mechanisms include inhibition of apoptosis, reduction of inflammation, protection of cardiomyocytes and endothelial function, promotion of angiogenesis, and synergistic lipid-lowering and plaque-stabilizing effects (Cheng et al.; Li et al., 2010; Wu et al., 2018; Yang et al., 2006; Yin, 2020; You et al., 2009; Zhang et al., 2020; Zhang Y. et al., 2008; Zhao et al., 2005; Zhu et al., 2008). With the growing number of publications and improved methodological quality of primary studies, a comprehensive systematic review and meta-analysis are warranted to evaluate the clinical effects and safety of TXL in AMI and to summarize its mechanisms of action.

**FIGURE 1:**
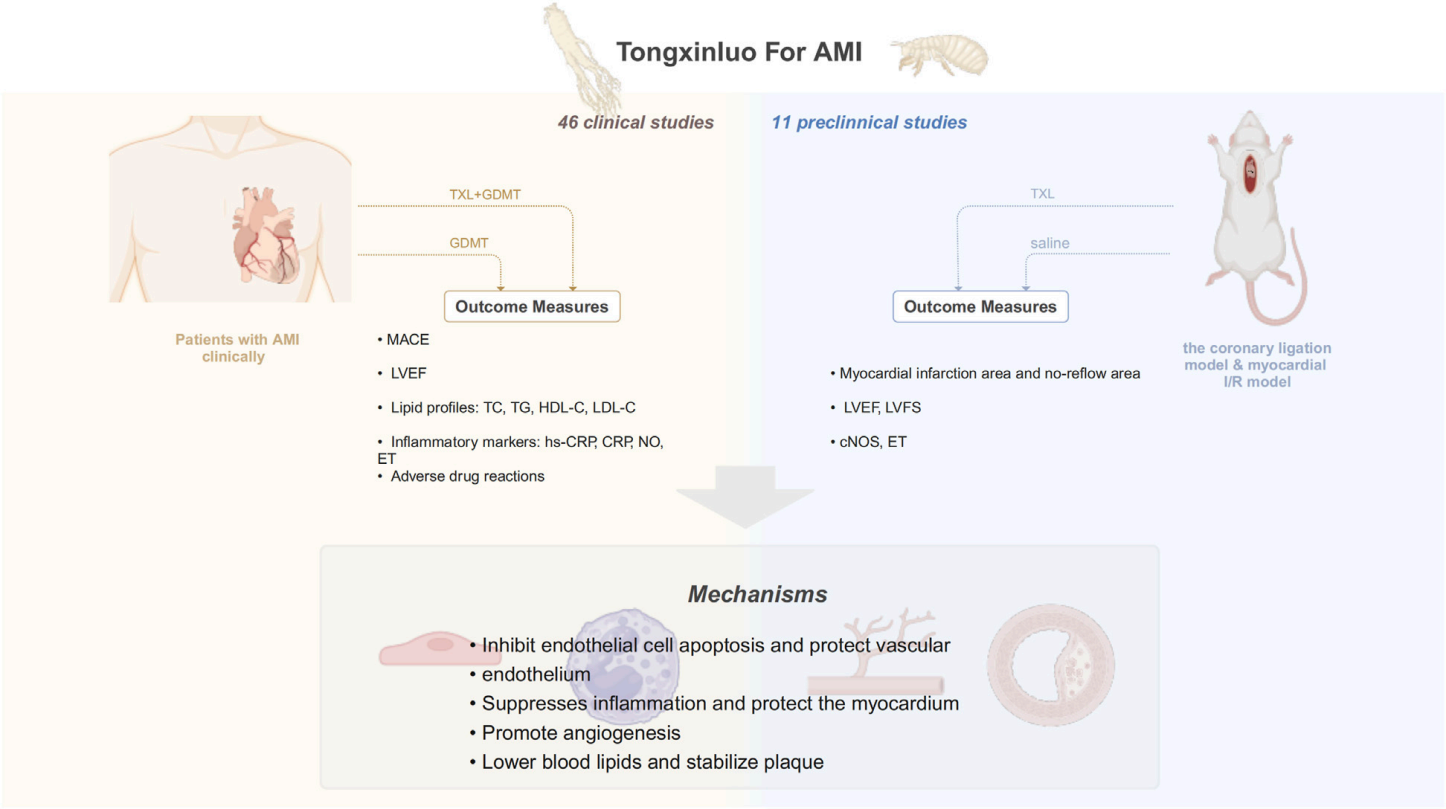
Graphical abstract.

This study aims to assess the effects and mechanisms of TXL in AMI based on current evidence from both clinical and animal studies. Specifically, it evaluates the clinical impact and safety of TXL as an adjunct to GDMT in AMI and examines the primary mechanisms through which TXL may exert its influence. A graphical abstract of the article is provided in Figure 1.

## 2 Materials and methods

### 2.1 Taxonomic validation of constituent species

All medicinal materials contained in Tongxinluo were taxonomically validated using the Medicinal Plant Names Services (MPNS) and the Catalogue of Life. The formulation comprises the following constituents: *Panax ginseng* C.A.Mey. [Araliaceae; Ginseng radix et rhizoma]; *Whitmania pigra* Whitman *or Hirudo nipponia* Whitman or *Whitmania acranulata* Whitman [Hirudinidae; Hirudo]; *Buthus martensii* Karsch [Buthidae; Scorpio]; *Paeonia lactiflora* Pall. or *Paeonia veitchii* Lynch [Paeoniaceae; Paeoniae Radix Rubra]; *Cryptotympana atrata* (Fabricius) [Cicadidae; Cicadidae Periostracum]; *Eupolyphaga sinensis* Walker or *Polyphaga plancyi* Bolívar [Corydiidae; Eupolyphaga seu Steleophaga]; *Scolopendra subspinipes mutilans* L. Koch [Scolopendridae; Scolopendra]; *Santalum album* L. [Santalaceae; Santali Albi Lignum]; *Dalbergia odorifera* T.C.Chen [Fabaceae; Dalbergiae Odoriferae Lignum]; *Boswellia carterii* Birdw. or *Boswellia sacra* Flück. [Burseraceae; Olibanum]; *Ziziphus jujuba Mill.var.spinosa* (Bunge) Hu ex H.F.Chou [Rhamnaceae; Ziziphi Spinosae Semen]; Borneolum Syntheticum [synthetic(±)-Borneol, CAS No. 507-70-0].

### 2.2 Headings

A systematic computer-based search was performed across eight databases (The Cochrane Library, PubMed, Web of Science, Embase, CNKI, WanFang Data, VIP, and SinoMed) and four clinical trial registries (ClinicalTrials.gov, WHO International Clinical Trials Registry Platform, Chinese Clinical Trial Registry, and International Traditional Medicine Clinical Trial Registry) to identify relevant randomized controlled trials (RCTs) and animal studies investigating the use of TXL in AMI. The search covered all publications from the inception of each database up to 1 April 2025. The search strategy incorporated a combination of subject headings and free-text terms. Additionally, manual searches of reference lists were conducted to identify additional eligible studies. The search was restricted to articles published in English or Chinese. The detailed search strategies and results for each database and registry are provided in the Supplementary Material.

### 2.3 Inclusion criteria

#### 2.3.1 Types of studies

Animal studies or RCTs.

#### 2.3.2 Participants

Clinical studies were required to include patients with a clinical diagnosis of AMI. Animal studies were required to use either coronary artery ligation or myocardial ischemia-reperfusion models.

#### 2.3.3 Interventions

In clinical trials, the trail group received guideline-directed medical therapy (GDMT) combined with TXL, while the control group received GDMT alone or GDMT combined with a placebo. In animal studies, the trail group received TXL at any dosage, and the control group received an equivalent volume of non-active vehicle (saline or distilled water) or no treatment.

#### 2.3.4 Outcome measures

Outcomes for clinical studies included: ①All-cause mortality, cardiovascular mortality, incidence of myocardial reinfarction, incidence of repeat revascularization, incidence of heart failure, incidence of angina pectoris, and incidence of arrhythmias; ② Left ventricular ejection fraction (LVEF); ③Lipid profiles: total cholesterol (TC), triglycerides (TG), high-density lipoprotein cholesterol (HDL-C), and low-density lipoprotein cholesterol (LDL-C); ④Inflammatory markers: hypersensitive C-reactive protein (hs-CRP), C-reactive protein (CRP), nitric oxide (NO), and endothelin-1 (ET-1); ⑤ Adverse drug reactions.

Outcomes for animal studies included: ① Myocardial infarction area and no-reflow area; ②Cardiac function: LVEF and left ventricular fractional shortening (LVFS); ③ Constitutive nitric oxide synthase (cNOS) and ET.

### 2.4 Exclusion criteria

1. Repeated studies, reviews, clinical protocols, comments, case reports, etc.
2. Studies that include other traditional Chinese medicines or related traditional Chinese medicine interventions apart from TXL.
3. Studies for which research data cannot be obtained even after contacting the original authors.
4. Studies without a control group.
5. Repeatedly published studies.
6. Clinical studies with a sample size of fewer than 50 cases.

### 2.5 Data extraction

Two reviewers (Yang Wu and Jingxue Guo) independently extracted data from the included studies. The following information was collected: (i) first author’s name and year of publication; (ii) characteristics of clinical patients or research animals], including age, sample size, intervention measures, as well as species, quantity, and weight for animal studies; (iii) methods used for establishing animal models and anesthesia protocols; (iv) outcome indicators, including clinical outcomes, inflammation-related markers and adverse drug reactions. If multiple time points were reported, the results from the final time point were selected.

### 2.6 Risk of bias in included studies

The risk of bias in clinical studies was assessed using the Cochrane RoB-2 tool, which evaluates the following domains: randomization process, deviations from intended interventions, missing outcome data, measurement of outcome, selection of the reported result and overall risk (Sterne et al., 2019). Two reviewers (Yang Wu and Jingxue Guo) independently performed the assessments. Discrepancies were resolved through discussion or consultation with the corresponding author (Xian Wang).

For animal studies, the SYRCLE’s risk of bias tool was applied. This tool comprises 10 items: sequence generation, baseline characteristics, allocation concealment, blinding of placement randomization, blinding of investigators, assessment of randomized outcomes, blinding of outcome evaluators, incomplete data, selective outcome reporting, other biases (Hooijmans et al., 2014). Each item was scored as 1 point if adequately reported.

### 2.7 Endpoint setting

In clinical studies, primary endpoints included all-cause mortality, cardiovascular mortality, and incidence of myocardial reinfarction. Secondary endpoints included incidence of revascularization, heart failure, angina, and arrhythmias; LVEF; lipid profiles: TC, TG, HDL-C, LDL-C; inflammatory markers: hs-CRP, CRP, NO, ET-1; and adverse effects.

In animal studies, primary endpoints were myocardial infarction area and no-reflow area. Secondary endpoints included LVEF, LVFS, cNOS, and ET.

### 2.8 Statistical analysis, subgroup analysis, and sensitivity analysis

Statistical analysis was performed using RevMan software (version 5.4). Heterogeneity was assessed using the I^2^ test. According to the Cochrane Handbook for Systematic Reviews of Interventions/Analysis of Heterogeneity, an I^2^ value ranging from 0% to 50% indicates low heterogeneity, justifying the use of a fixed-effects model. Conversely, an I^2^ value between 50% and 100% indicates high heterogeneity, necessitating the use of a random-effects model (Higgins et al., 2019). P < 0.05 is considered statistically significant. Relative risk (RR) is used as the effect measure for count data, while mean difference (MD) or standardized mean difference (SMD) is used as the effect measure for measurement data.

Where substantial heterogeneity was detected, sensitivity or subgroup analyses were performed to explore potential sources.

### 2.9 Publication bias

Publication bias was evaluated using funnel plots, Egger’s test, and Duval and Tweedie’s trim-and-fill method, implemented in R, to impute potentially missing studies and estimate adjusted effect sizes.

### 2.10 Protocol registration

The protocol registration for this systematic review was completed on INPLASY® (www.inplasy.com) under the number INPLASY202580092.

### 2.11 Assessment of certainty of evidence

The overall certainty of evidence for each outcome was rated using the GRADE approach. Initial certainty for outcomes from RCTs started as high but was downgraded based on the following criteria: risk of bias, inconsistency, indirectness, imprecision, and publication bias. Two authors (Yang Wu and Jingxue Guo) independently performed GRADE assessments, with disagreements resolved by consensus or third-author arbitration (Xian Wang).

## 3 Results

### 3.1 Included literature

**FIGURE 2:**
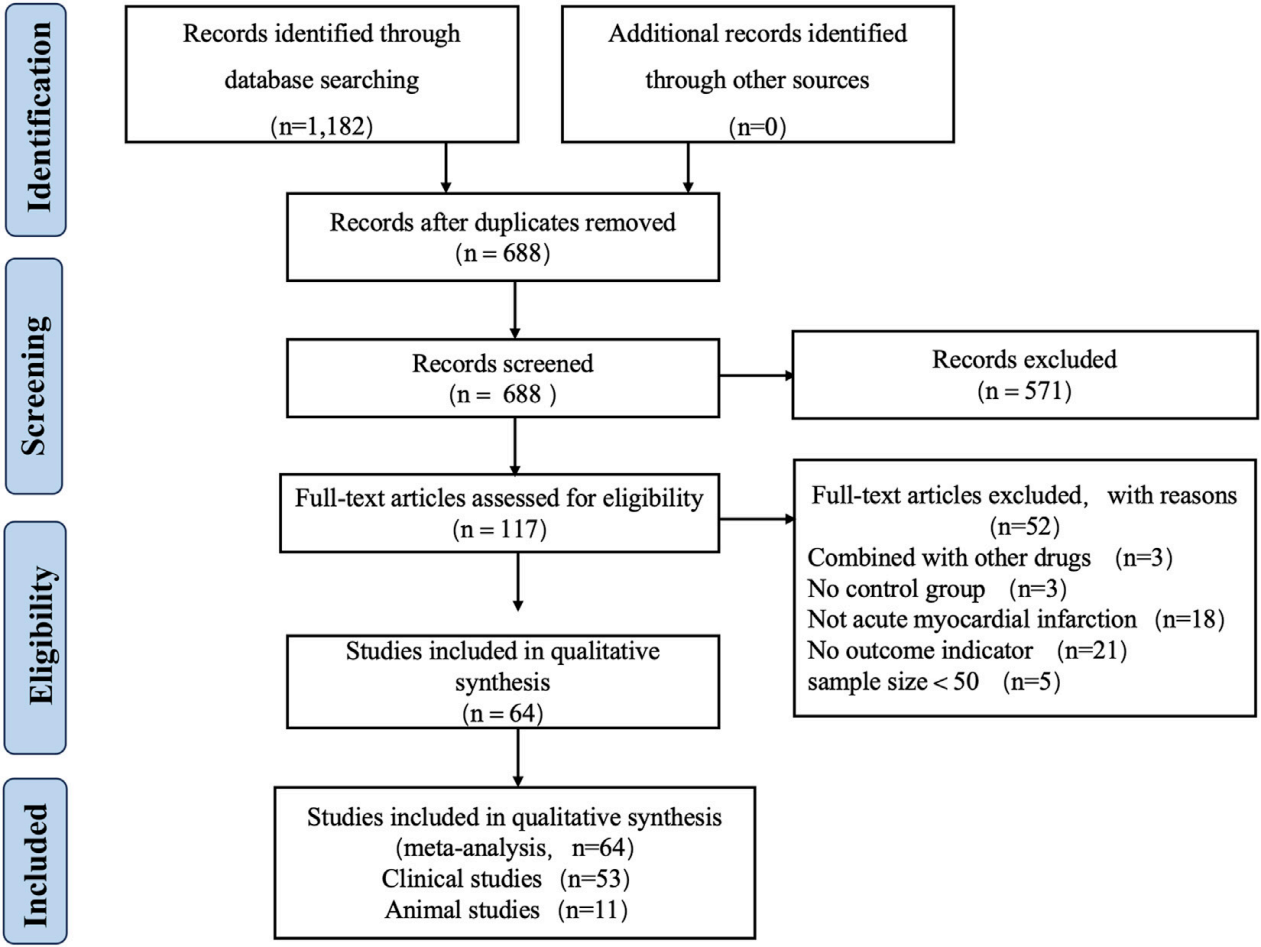
Literature screening process and results.

A total of 1,182 articles were retrieved from eight databases, and 688 articles remained after removing duplicates using Endnote X9 software. Two reviewers (Yang Wu and Jingxue Guo) independently screened the titles and abstracts, resulting in the selection of 117 articles. Two reviewers then thoroughly reviewed the full texts of these 117 articles, which ultimately included 54 clinical studies and 11 animal studies published between 2005 and 1 April 2025. Any disagreements during the review process were resolved by Xian Wang. Figure 2 illustrates the flowchart of the study inclusion process.

### 3.2 Basic characteristics of included studies

#### 3.2.1 Basic characteristics of clinical studies

Among the 54 clinical trials included in this review, 50 were published in Chinese journals and 4 in English journals. The studies were published between 2005 and 1 April 2025, and sample sizes ranged from 51 to 3,777 participants. In the trial group, the regimen was TXL combined with GDMT. The control group received either GDMT alone or GDMT combined a placebo. The dosage of TXL was usually 2-5 capsules, 3 times a day. The duration of treatment varied, with most studies reporting a duration of 1–12 months (Table 1).

**TABLE 1:** Basic characteristics of 54 clinical studies.

Study id	Gander (male/female)		Age		Sample size (T/C)	Disease	T	C	Usage and dosage, duration	Outcomes
	T	C	T	C						
Yang, YJ 2023	1,456/433	1,448/440	61.4 ± 12.1	61.5 ± 12.1	3,777 (1889/1888)	STEMI	TXL plus GDMT	GDMT plus Placebo	Pre-PCI: 8 capsules, tid; post-PCI: 4 capsules, tid; 12 months	①②⑥
Meng, XX 2023	24/22	26/19	52.06 ± 9.69	51.28 ± 10.03	91 (46/45)	AMI, post-PCI	TXL plus GDMT	GDMT	3 capsules, tid; 12 weeks	①②⑤
Ren, FB 2023	14/31	20/25	56.27 ± 6.68	56.31 ± 6.34	90 (45/45)	AMI	TXL plus GDMT	GDMT	5 capsules, tid; 2 weeks	①②③⑤
Li, YH 2023	16/14	15/15	61.05 ± 14.85	60.88 ± 15.09	60 (30/30)	AMI	TXL plus GDMT	GDMT	4 capsules, tid; 8 weeks	①②④
Zhou, CJ 2022	21/13	20/14	49.82 ± 6.18	50.09 ± 5.86	68 (34/34)	AMI, perioperative period	TXL plus GDMT	GDMT	2-4 capsules, tid; 4 weeks	②⑤
Mai, QX 2022	21/14	22/13	59.47 ± 8.65	59.59 ± 8.36	70 (35/35)	AMI	TXL plus GDMT	GDMT	2 capsules, tid; 1 week	③⑤
Yu, YM 2021	29/17	28/18	58.5 ± 5.0	57.9 ± 4.8	92 (46/46)	AMI, post-PCI	TXL plus GDMT	GDMT	3 capsules, tid; 12 weeks	③⑥
Shi, ML 2021	23/19	22/20	53.12 ± 8.95	52.36 ± 10.48	84 (42/42)	AMI	TXL plus GDMT	GDMT	4 capsules, tid; 15 days	③
Huang, J 2021	26/15	26/16	65.24 ± 7.11	65.52 ± 7.25	83 (41/42)	AMI	TXL plus GDMT	GDMT	5 capsules, tid; 8 weeks	③⑤⑥
Hong, ZZ 2021	30/27	28/29	63.05 ± 5.72	62.58 ± 5.65	114 (57/57)	AMI, post-PCI	TXL plus GDMT	GDMT	4 capsules, tid; 12 weeks	③⑥
Wang, WW 2021	31/19	33/17	57.23 ± 8.92	56.70 ± 9.42	100 (50/50)	AMI, post-PCI	TXL plus GDMT	GDMT	4 capsules, tid; 4 weeks	③⑤
Jia, HL 2021	22/23	25/20	45.39 ± 7.02	44.82 ± 6.73	90 (45/45)	AMI	TXL plus GDMT	GDMT	2 capsules, tid; 4 weeks	③⑥
Shen, Q 2021	29/21	27/23	59.14 ± 5.33	59.23 ± 5.41	100 (50/50)	AMI	TXL plus GDMT	GDMT	5 capsules, tid; 2 weeks	③⑤⑥
Yu, ZL 2021	31/29	36/24	55.98 ± 10.04	57.65 ± 9.76	120 (60/60)	AMI, post-PCI	TXL plus GDMT	GDMT	2-4 capsules, tid; 8 weeks	⑥
Zhao, XP 2021	39/14	38/15	51–64	50–62	106 (53/53)	AMI	TXL plus GDMT	GDMT	2 capsules, tid; 2 weeks	⑥
Zhao, YY 2020	40/32		61.36 ± 12.19		72 (38/34)	AMI, post-PCI	TXL plus GDMT	GDMT	Post-PCI: 8 tablets; post-PCI: 4 capsules, tid; 4 weeks	①③⑥
Ge, ZQ 2020	35/25	38/22	42.7 ± 5.2	42.2 ± 5.7	120 (60/60)	AMI, post-PCI	TXL plus GDMT	GDMT	4 capsules, tid; 24 weeks	①②⑤
Liu, H 2020	54/53	58/49	59.6 ± 18.5	57.1 ± 15.3	214 (107/107)	AMI, post-PCI	TXL plus GDMT	GDMT	3 capsules, tid; 12 weeks	③
Ding, N 2020	28/15	25/18	56.11 ± 6.23	56.37 ± 6.49	86 (43/43)	AMI	TXL plus GDMT	GDMT	4 capsules, tid; 2 weeks	③⑤
Zhou, LY 2019	29/18	27/20	56.87 ± 6.59	57.42 ± 7.23	94 (47/47)	AMI	TXL plus GDMT	GDMT	4 capsules, tid; 4 weeks	③⑤
Chen, XY 2019	29/23	30/22	66.32 ± 3.28	67.82 ± 3.74	104 (52/52)	AMI	TXL plus GDMT	GDMT	4 capsules, tid; 12 weeks	③
Xu, WW 2019	16/23	17/22	71.88 ± 5.98	70.56 ± 6.48	78 (39/39)	AMI, post-PCI	TXL plus GDMT	GDMT	2-4 capsules, tid; 4 weeks	②
Wang, CL 2018	30/16	28/18	52.51 ± 12.66	51.16 ± 11.54	92 (46/46)	AMI	TXL plus GDMT	GDMT	4 capsules, tid; 12 weeks	③⑤
Zhou, S 2018	29/18	27/20	56.94 ± 4.61	57.38 ± 5.13	94 (47/47)	AMI	TXL plus GDMT	GDMT	5 capsules, tid; 2 weeks	③⑤
Peng, ZP 2017	43/42	45/40	61.3 ± 3.8	62.8 ± 3.5	170 (85/85)	AMI, Post-PCI	TXL plus GDMT	GDMT	4 capsules, tid; 1 week	①②
Wang, YL 2016	19/11	18/12	58 ± 7	58 ± 6	60 (30/30)	AMI, post-PCI	TXL plus GDMT	GDMT	4 capsules, tid; 48 weeks	③⑤
Chen, ZQ 2016	28/12	26/14	60.5 ± 14.6	61.7 ± 13.6	80 (40/40)	AMI, post-PCI	TXL plus GDMT	GDMT	4 capsules, tid; 1 week	①②③⑤
Tian, ZT 2014	22/8	19/11	54.9 ± 10.4	54.5 ± 9.8	60 (30/30)	AMI, post-PCI	TXL plus GDMT	GDMT	4 capsules, tid; 12 weeks	①②③⑤
Dong, SQ 2012	45/18		40–77		63 (35/28)	AMI, post-PCI	TXL plus GDMT	GDMT	4 capsules, tid; 48 weeks	①⑤
Wang, HZ 2012	38/24		71.60 ± 4.20		62 (31/31)	AMI	TXL plus GDMT	GDMT	2 capsules, tid; 20 days	①②③
Yang, W 2012	17/13	18/11	64 ± 11	66 ± 12	59 (30/29)	AMI, post-PCI	TXL plus GDMT	GDMT	3 capsules, tid; 12 weeks	③
Kuang, YD2011	75/35		56.34 ± 10.82		110 (60/50)	STEMI	TXL plus GDMT	GDMT	3 capsules, tid; 30 days	③⑤
You, MS 2011	83/8		54 ± 5.6	47 ± 5.2	91 (45/46)	AMI	TXL plus GDMT	GDMT	4 capsules, tid; 8 weeks	③
Liao, CL 2010	21/18	20/17	60.3 ± 9.9	64.3 ± 11.7	76 (39/37)	AMI	TXL plus GDMT	GDMT	3 capsules, tid; 2 weeks	③⑤
Huang, B 2010	68/52		58.3 ± 12.6		120 (62/58)	AMI, post-PCI	TXL plus GDMT	GDMT	4 capsules, tid; 24 weeks	①④⑤
Liang, YM 2010	52/28		40–70		80 (42/38)	AMI, post-PCI	TXL plus GDMT	GDMT	2 capsules, tid; 24 weeks	①②④
Yang, W 2009	22/8	25/4	58	56	59 (30/29)	AMI, post-PCI	TXL plus GDMT	GDMT	3 capsules, tid; 12 weeks	①②③
Zhang, XP 2009	70/26	57/25	60 ± 5	58 ± 8	178 (96/82)	AMI	TXL plus GDMT	GDMT	3 capsules, tid; 96 weeks	①②⑥
Zhao, QH 2009	40/10	35/13	59.6	60.3	98 (50/48)	AMI	TXL plus GDMT	GDMT	3 capsules, tid; 48 weeks	①②
Fan, SM 2008	50/11		56.23 ± 12.06		61 (34/27)	AMI, post-PCI	TXL plus GDMT	GDMT	NT; 4 weeks	④
Chen, W 2008	23/12	25/10	68 ± 7	69 ± 7	70 (35/35)	AMI	TXL plus GDMT	GDMT	3 capsules, tid; 6 weeks	③
Wang, G 2007	31/3	30/4	58.08 ± 11.14	57.69 ± 11.18	68 (34/34)	AMI, post-PCI	TXL plus GDMT	GDMT	4 capsules, tid; 24 weeks	③
Chen, H 2007	32/38		58 ± 11		60 (30/30)	AMI, post-PCI	TXL plus GDMT	GDMT	2-4 capsules, tid; 8 weeks	①②③④⑤
Li, ZX 2006	21/9	20/10	48–73	49–75	60 (30/30)	AMI	TXL plus GDMT	GDMT	3 capsules, tid; 3 weeks	④⑥
You, SJ 2005	52/8	40/12	57.08 ± 11.04	58.74 ± 11.24	112 (60/52)	AMI	TXL plus GDMT	GDMT	4 capsules, tid; 24 weeks	③
Zhang, JW 2006	43/18		64 ± 9		61 (30/31)	AMI, post-PCI	TXL plus GDMT	GDMT	3 capsules, bid; 2 weeks	⑤
Shen, X 2024	35/28	40/23	53.09 ± 12.33	52.35 ± 11.42	126 (63/63)	STEMI, post-PCI	TXL plus GDMT	GDMT	3 capsules, tid; 4 weeks	⑤
Adili2024	23/17	20/20	60.45 ± 4.58	59.56 ± 5.17	80 (40/40)	AMI, post-PCI	TXL plus GDMT	GDMT	2 capsules, tid; 24 weeks	①②③⑤
Li, P 2024	53/44	56/41	55.87 ± 8.46	55.83 ± 8.42	194 (97/97)	AMI, post-PCI	TXL plus GDMT	GDMT	2-4 capsules, tid; 8 weeks	①②③⑤
Xiong, L 2024	22/18	23/17	71.86 ± 8.15	71.49 ± 8.32	80 (40/40)	AMI, post-PCI	TXL plus GDMT	GDMT	4 capsules, tid; 12 weeks	①②③
Xu, YG 2024	27/19	25/21	58. 55 ± 6. 81	58. 42 ± 6. 75	92 (46/46)	AMI, post-PCI	TXL plus GDMT	GDMT	4 capsules, tid; 4 weeks	③⑤⑥
Yan, DY 2024	20/13	19/14	0.34 ± 5.28	59.63 ± 5.14	66 (33/33)	AMI	TXL plus GDMT	GDMT	5 capsules, tid; 2 weeks	⑥
Zhang, XP 2024	29/24	28/25	62.82 ± 5.98	63.25 ± 5.77	106 (53/53)	AMI, post-PCI	TXL plus GDMT	GDMT	4 capsules, tid; 4 weeks	③⑤
Yan, S 2024	26/19	25/20	58.85 ± 5.87	59.52 ± 6.58	90 (45/45)	AMI, post-PCI	TXL plus GDMT	GDMT	4 capsules, tid; 1 year	③⑤

Note: T, trail group; C, control group; AMI, acute myocardial infarction; STEMI, ST-segment elevation myocardial infarction; TXL, Tongxinluo capsule; GDMT, guideline-directed medical therapy; NT, not mentioned. ①All-cause mortality, cardiovascular mortality, incidence of myocardial reinfarction; ② Incidence of repeat revascularization, heart failure, angina pectoris, arrhythmias; ③ LVEF; ④ Lipid profiles; ⑤ Inflammatory markers; ⑥ Adverse drug reactions.

#### 3.2.2 Basic characteristics of animal studies

A total of 11 animal studies were included, of which 8 studies were published in Chinese journals and 3 studies were published in English journals. Regarding anesthesia methods, 10 studies specified the types of anesthesia used, while 1 study did not provide details. The modeling approaches primarily were mainly coronary artery ligation models and AMI reperfusion models. All trail groups received different doses of TXL, while control groups received 0.9% sodium chloride or drinking water. The duration of all experiments ranged from 1 day to 4 weeks (Table 2).

**TABLE 2:** Basic characteristics of 11 animal studies.

Study ID	Model	Species (sex, weight)	Anesthetic	T (dose, sample size)	C (dose, sample size)	Duration	Effect indexes
Zhang, HT 2020	AMI Reperfusion Model	Chinese Miniature Pigs (sex-matched, 20–30 kg)	Azaperone combined with thiopental sodium	TXL (administered at 0.4 g/kg 90 min before coronary artery ligation, followed by 0.2 g/kg/day for 7 days post-surgery, *n =* 8)	No Intervention (*n =* 8)	Assessment at 7 Days Post-Surgery	⑥
Yin, YJ 2019	Coronary artery ligation	Male rats (200–220 g)	Intraperitoneal injection of 10% chloral hydrate (3.5 mL/kg)	TXL (0.4 g/kg/d, *n =* 10)	Administration of sodium carboxymethylcellulose solution (3 mL/kg, *n =* 10)	Postoperative observation period of 4 weeks	①②
Wu, HT 2018	Coronary external application of 70% iron chloride	Rabbits (both male and female, 2.5–3.0 kg)	25% ethyl carbamate (4 mL/kg)	TXL (0.125 g/kg/d, *n =* 8)	0.9% NaCl (*n =* 8)	7 days	①②
Li, XD 2010	AMI Reperfusion Model	Chinese Miniature Pigs (both male and female, 20–30 kg)	Mixed anesthesia with 25 mg/kg ketamine and 1 mg/kg diazepam	TXL (0.05 g/kg, *n =* 8)	0.9NaCl (15 mL, *n =* 8)	1 dose (1 h before surgery)	③④⑤
You, MS 2009	Coronary artery ligation	Rats (male, 190–210 g)	Intraperitoneal injection of 10% chloral hydrate	TXL (1 g/kg/d, *n =* 13)	0.9NaCl (*n =* 12)	4 weeks post-operation	①②③
Cheng, YT 2009	AMI Reperfusion Model	Chinese miniature pigs (both sexes, 20–30 kg)	Mixed anesthesia with ketamine and Diazepam	TXL (0.4 g/kg, *n =* 7)	drinking water (30 mL, *n =* 7)	1 dose (3 h before surgery)	③④⑤
Zhu, HM 2008	Coronary artery ligation	Healthy mixed-breed dogs (12–15 kg, both sexes)	Intravenous injection of Sodium Pentobarbital at 30 mg/kg	TXL (0.36 g/kg, *n =* 5)	*n =* 5	1 dose (30 min after surgery)	③
Zhang, YF 2008	Coronary artery ligation	Rats (both sexes, 160–180 g)	20% Urethane solution	TXL (0.6 g/kg/d, *n =* 12)	0.9% NaCl (10 mL/kg/d, *n =* 12)	14 days	③
Yang, YJ 2006	AMI Reperfusion Model	Chinese miniature pigs (both sexes, 30 kg)	Not mentioned	TXL (0.2 g/kg/d, *n =* 8)	*n =* 8	3 days prior to surgery	③④
Yang, YJ 2006	Coronary artery ligation	Large white rabbits (males, 2.5–3 kg)	Intramuscular injection of Ketamine at 100 mg/kg	TXL (1 g/kg/d, *n =* 8)	*n =* 8	3 days prior to surgery	③⑥
Zhao, JL 2005	AMI Reperfusion Model	Chinese miniature pigs (both sexes, 27–33 kg)	Intramuscular injection of Ketamine at 100 mg/kg	TXL (0.2 g/kg/d, *n =* 8)	0.9NaCl (*n =* 8)	3 days prior to surgery	③④⑤⑥

Note: ①: LVEF; ②: LVFS; ③: Myocardial infarction area; ④: no-reflow area; ⑤: cNOS; ⑥: ET.

### 3.3 Assessment of risk of bias

#### 3.3.1 Risk of bias in clinical studies

**FIGURE 3:**
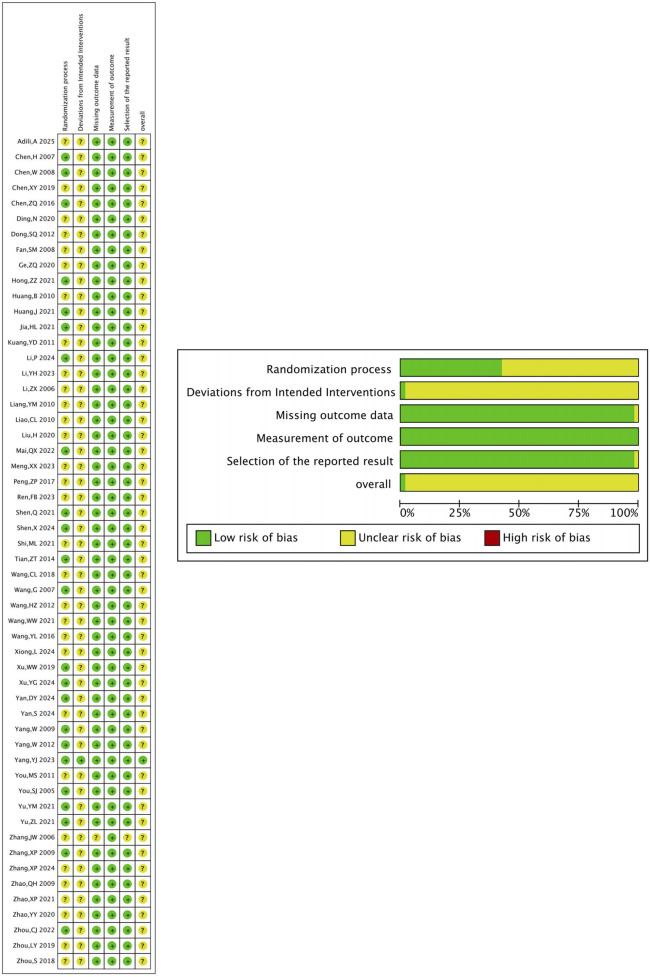
Methodological assessment of included clinical studies (RoB-2 method). **(A)** Risk of bias graph. **(B)** Risk of bias summary.

The risk of bias of 54 clinical studies was assessed using the RoB-2 tool. Of these studies, 23 reported specific randomization methods, and 3 used double blinding. Most studies had complete outcome data and did not register clinical trial protocols. No additional bias was identified in these 54 studies (Figure 3).

#### 3.3.2 Risk of bias in animal studies

Eleven animal experiments were assessed using the SYRCLE risk of bias tool. Of these, 2 studies had a low risk of random sequence generation. All animal studies had comparable baseline characteristics and complete outcome data. Most studies had no additional bias, and some outcome measures were randomly selected (Table 3).

**TABLE 3:** Methodological assessment of included animal studies (SYRCLE Tool).

Study id	A	B	C	D	E	F	G	H	I	J	Overall score
Zhang, HT 2020	0	1	0	0	1	0	1	1	1	1	6
Yin, YJ 2019	0	1	0	0	1	0	1	1	1	0	5
Wu, H T 2018	0	1	0	0	1	1	1	1	0	1	6
Li, X D 2010	0	1	0	0	1	0	1	1	1	0	5
You, MS 2009	0	1	0	0	1	1	1	1	1	1	7
Cheng, Y T 2009	0	1	0	0	1	0	1	1	1	1	6
Zhu, HM 2008	0	1	0	0	1	0	1	1	1	1	6
Zhang, YF 2008	0	1	0	0	1	0	1	1	0	1	5
Yang, YJ 2006	1	1	0	0	1	1	1	1	0	1	7
Yang, YJ 2006	0	1	0	0	1	0	1	1	1	1	6
Zhao, JL 2005	1	1	0	0	1	0	1	1	1	0	6

Note: A: Random sequence generation; B: Baseline characteristics; C: Allocation concealment; D: Randomization blinding; E: Blinding; F: Randomized outcome assessment; G: Blinding of outcome assessors; H: Incomplete data; I: Selective outcome reporting; J: Other biases.

### 3.4 Meta results of clinical studies

A total of 54 clinical studies were included in this review, which systematically evaluated the clinical effects of TXL in AMI and assessed the safety of the drug. The results of the clinical studies showed that TXL was associated with a reduction in the incidence of MACE, improved cardiac function, improved lipids, reduced inflammation markers and improved vascular endothelial function (P < 0.05). TXL also had fewer adverse effects including gastrointestinal reactions, bleeding, and dizziness and headaches.

#### 3.4.1 Primary MACE events

**FIGURE 4:**
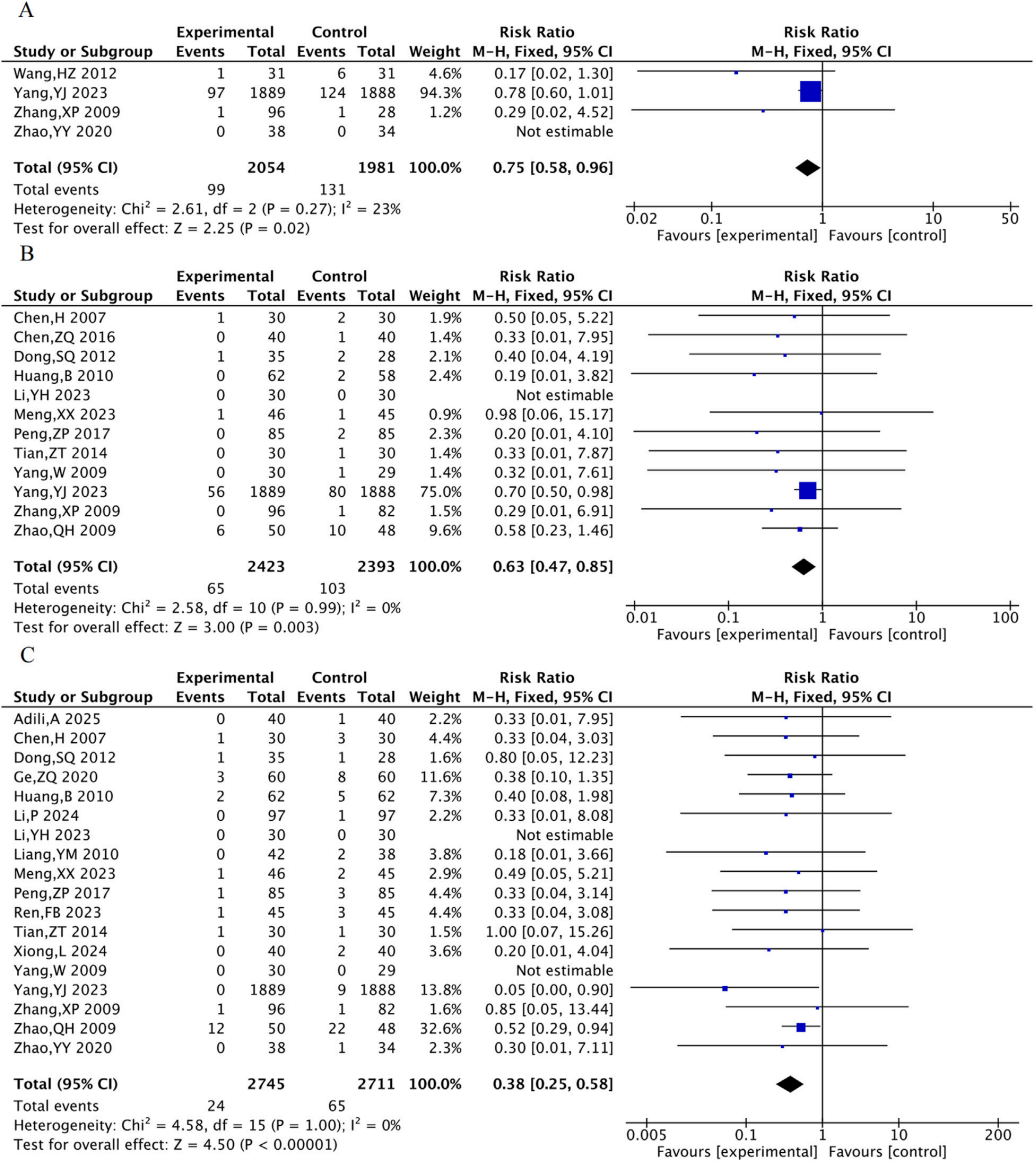
The meta-analysis result of three primary MACE events. **(A)** All-cause mortality. **(B)** Cardiovascular mortality. **(C)** Incidence of myocardial reinfarction.

Four RCTs were included for all-cause mortality, 12 for cardiovascular mortality, and 18 for the incidence of myocardial reinfarction. The results suggested that, compared with the control group, the combined TXL group had lower all-cause mortality [RR = 0.75, 95% CI (0.58, 0.96), P = 0.02], cardiovascular mortality [RR = 0.63, 95% CI (0.47, 0.85), P = 0.003] and the incidence of myocardial reinfarction [RR = 0.38, 95% CI (0.25, 0.58), P < 0.001] (Figure 4).

#### 3.4.2 Other MACE events

**FIGURE 5:**
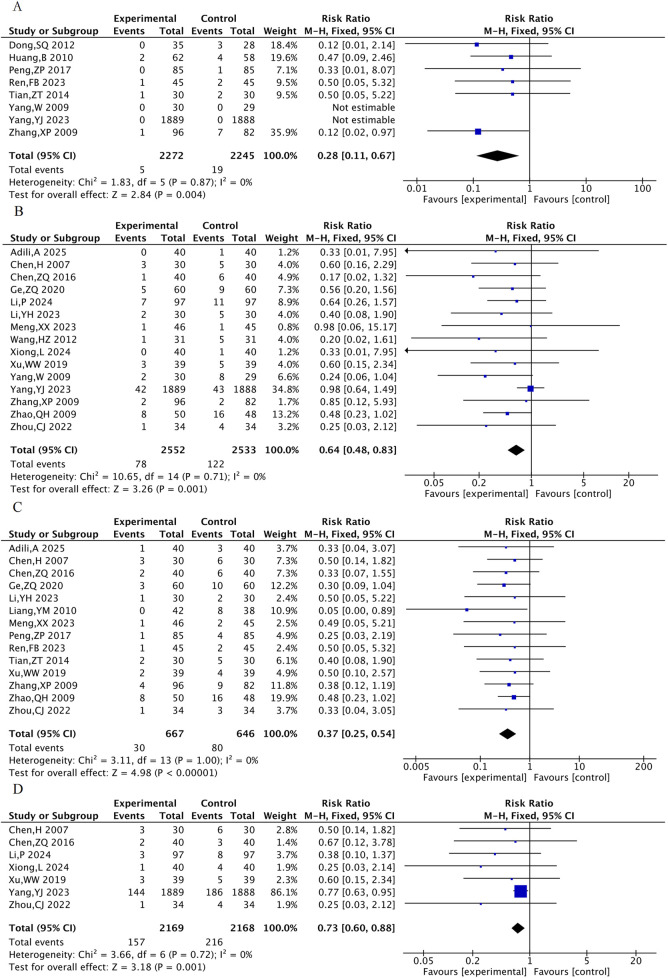
The meta-analysis result of the other MACE events. **(A)** Incidence of repeated revascularization. **(B)** Incidence of heart failure. **(C)** Incidence of angina pectoris. **(D)** Incidence of arrhythmia.

Eight RCTs were included for the rate of revascularization, 15 RCTs for the rate of heart failure, 14 RCTs for the rate of angina and 7 RCTs for the rate of arrhythmia. The results showed that, compared with the control group, the TXL group had a lower incidence of revascularization [RR = 0.28, 95% CI (0.11, 0.67), P = 0.004], incidence of heart failure [RR = 0.64, 95% CI (0.48, 0.83), P = 0.001], incidence of angina pectoris [RR = 0.37, 95% CI (0.25, 0.54), P < 0.001], and incidence of arrhythmia [RR = 0.73, 95% CI (0.60, 0.88), P = 0.001] (Figure 5).

#### 3.4.3 Cardiac function indicator

**FIGURE 6:**
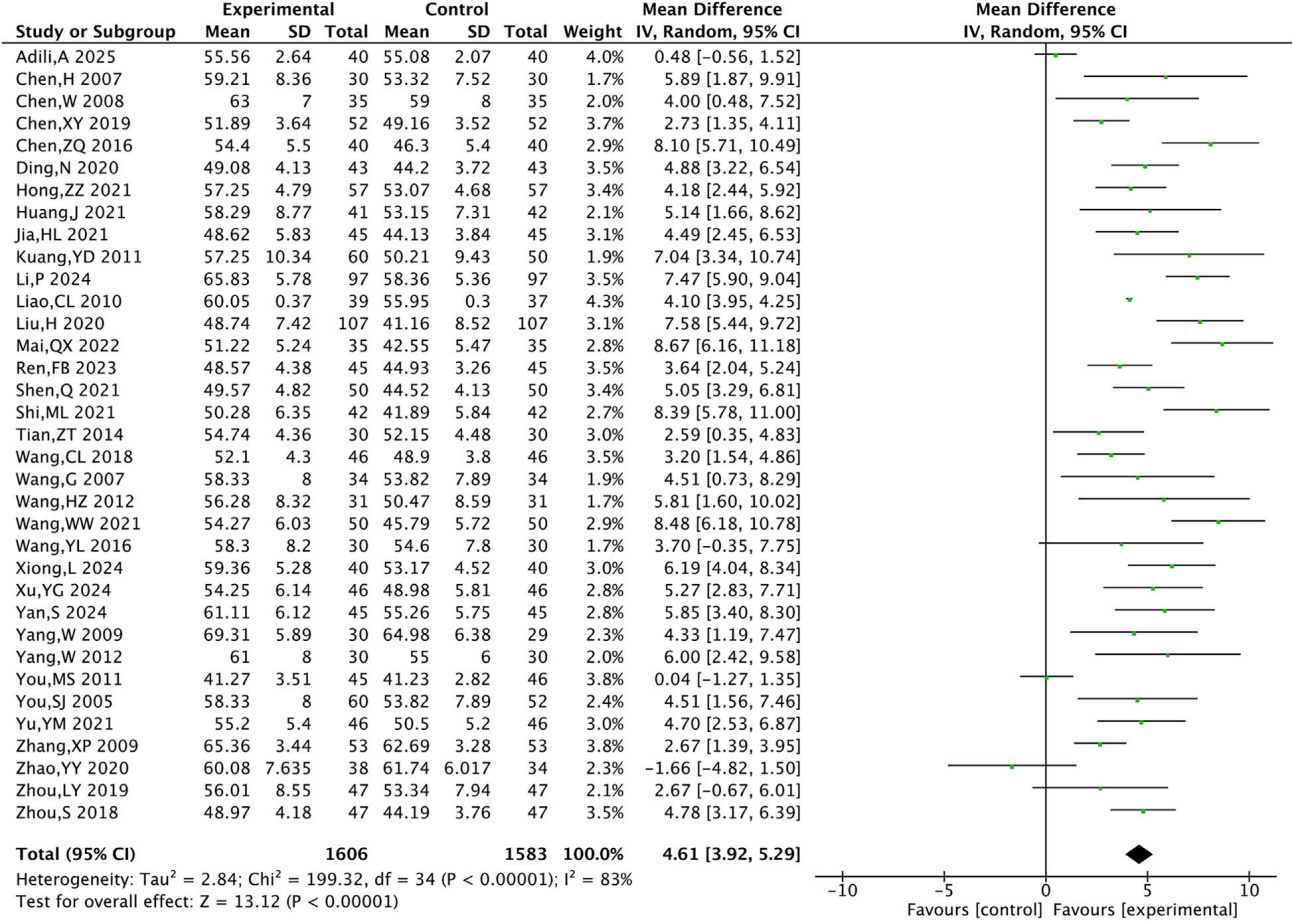
The meta-analysis result of LVEF.

A total of 35 RCTs including 3,189 patients were included for the cardiac function indicator LVEF. The meta-analysis showed that LVEF was higher in the TXL group compared with the control group [MD = 4.61, 95% CI (3.92, 5.29), P < 0.001](Figure 6).

#### 3.4.4 Lipid profile

**FIGURE 7:**
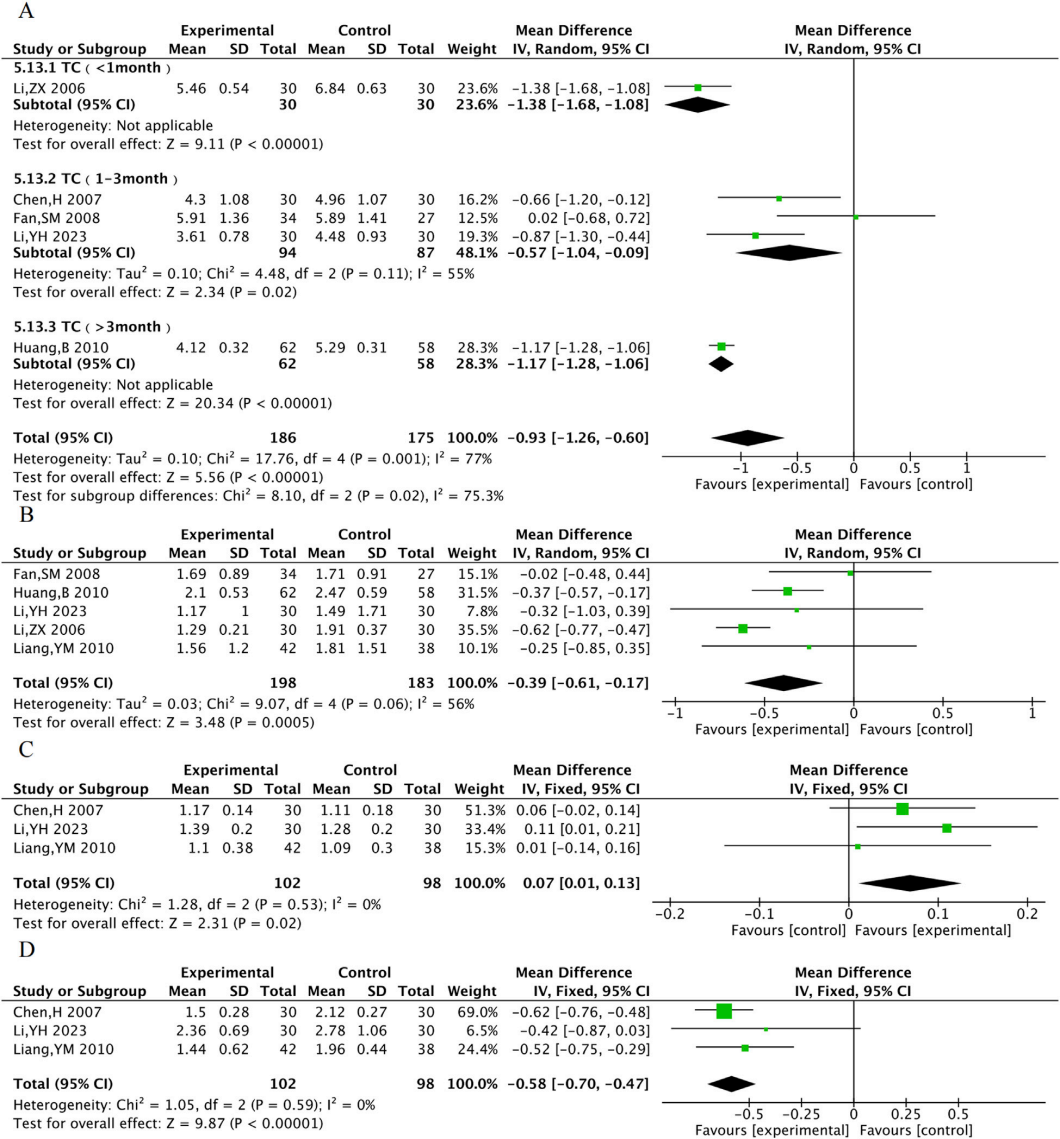
The meta-analysis result of TC **(A)**, TG **(B)**, HDL-C **(C)**, LDL-C **(D)**.

TC was assessed in 5 RCTs, TG in 5 RCTs, HDL-C in 3 RCTs, and LDL-C in 3 RCTs. The results showed that compared with the control group, the TXL combination group had lower TC [MD = −0.93, 95% CI (−1.26, −0.60), P < 0.001], TG [MD = −0.39, 95% CI (−0.61, −0.17), P < 0.001], LDL-C [MD = −0.58, 95% CI (−0.70, −0.47), P < 0.001], and higher HDL-C [MD = 0.07, 95% CI (0.01, 0.13), P = 0.02]. Due to the high degree of heterogeneity in TC, subgroup analyses were performed according to the time of administration. Heterogeneity within the 1–3 months subgroup decreased (P = 0.11, I^2^ = 55%), suggesting more stable improvements in TC after 1–3 months of TXL treatment. ([MD_TC 1–3month_ = −0.57, 95% CI (−1.04, −0.09), P = 0.02]) (Figure 7).

#### 3.4.5 Inflammation-related indicators

**FIGURE 8:**
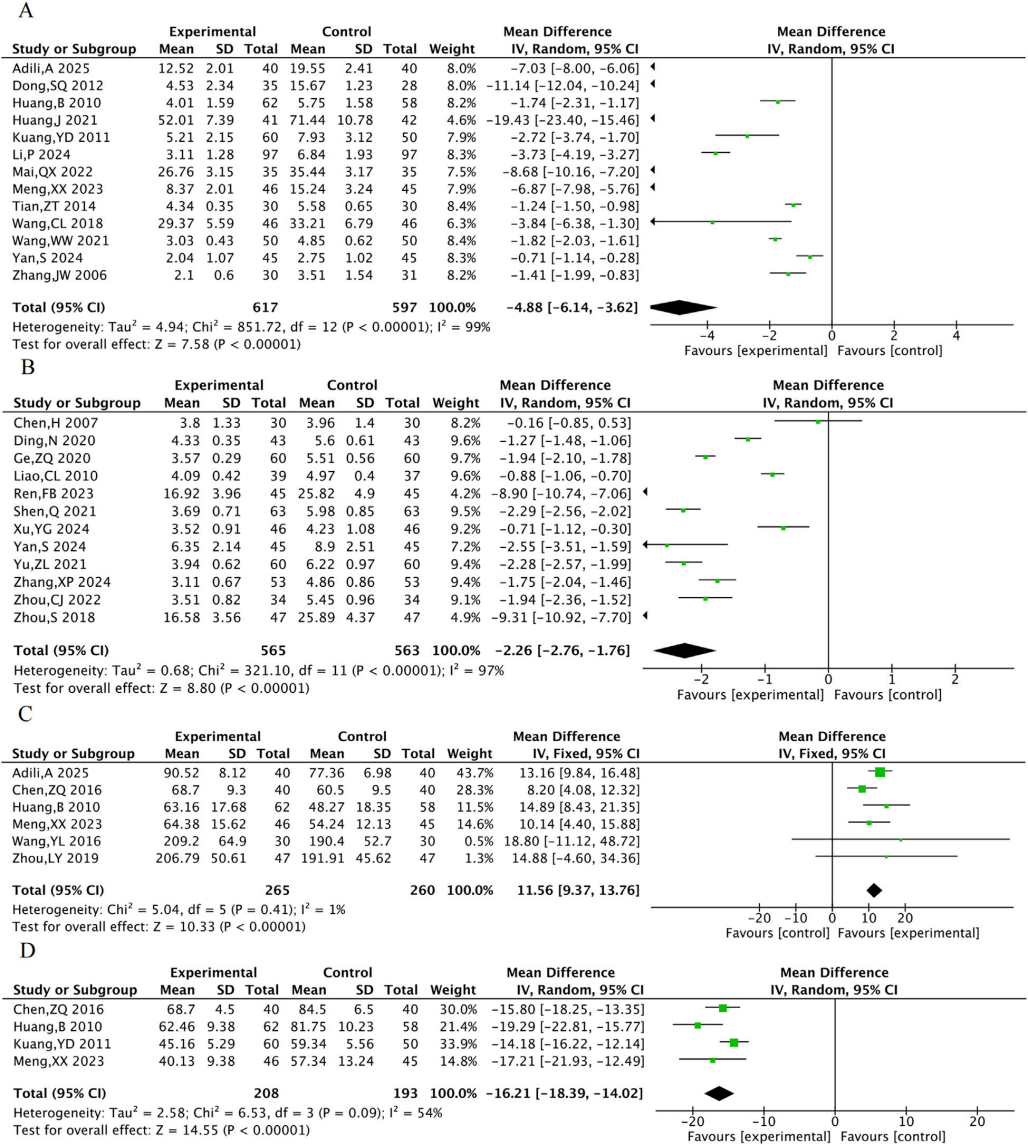
The meta-analysis result of hs-CRP **(A)**, CRP **(B)**, NO **(C)**, ET-1 **(D)** in AMI.

A total of 13 RCTs were included for hs-CRP, 12 for CRP, 6 for NO, and 4 for ET-1. The results indicated that compared with the control group, the combined TXL group had lower hs-CRP [MD = −4.88, 95% CI (−6.41, −3.62), P < 0.001], lower CRP [MD = −2.26, 95% CI (−2.76, −1.76), P < 0.001], lower ET-1 [MD = −16.21, 95% CI (−18.39, −14.02), P < 0.001] (Figure 8), and higher NO [MD = 11.56, 95% CI (9.37, 13.76), P < 0.001].

#### 3.4.6 Adverse drug reactions

**FIGURE 9:**
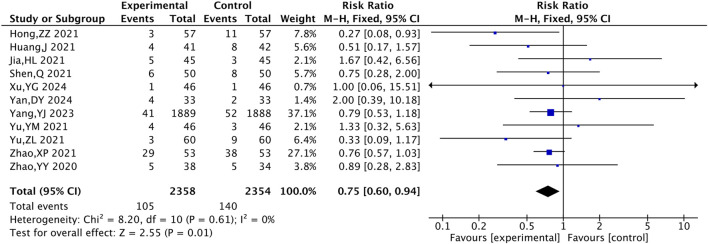
The meta-analysis result of total adverse drug reactions.

A total of 11 studies reported the adverse reactions of the two groups. The results showed that the TXL combination group experienced significantly fewer adverse reactions than the control group (P = 0.01) (Figure 9). Of the 11 studies, three specific adverse reactions were identified: gastrointestinal reactions, bleeding, and dizziness/headache. Meta-analyses of all 3 adverse reactions showed P > 0.05 for each type, indicating that there was no statistically significant difference between the two groups of 3 adverse reactions (Table 4).

**TABLE 4:** Meta-analysis of adverse drug reactions for each drug.

Types of adverse reactions	Number of incidences/overall sample size in T	Number of incidences/overall sample size in C	P
Gastrointestinal adverse reactions	18/356	23/353	P = 0.4
Bleeding	9/229	17/226	P = 0.11
Dizziness and headache	2/141	4/141	P = 0.45

Note: T, trail group; C, control group.

### 3.5 Meta results of animal studies

This study included a total of 11 animal studies and performed a meta-analysis of several outcome measures: myocardial infarction area, no-reflow area, cardiac function (LVEF and LVFS), cNOS, and ET. The analysis showed that compared to the control group, TXL was associated with lower myocardial infarction area and no-reflow area, and better cardiac function, endothelial and vascular function (P < 0.05).

#### 3.5.1 Myocardial infarction area and no-reflow area

**FIGURE 10:**
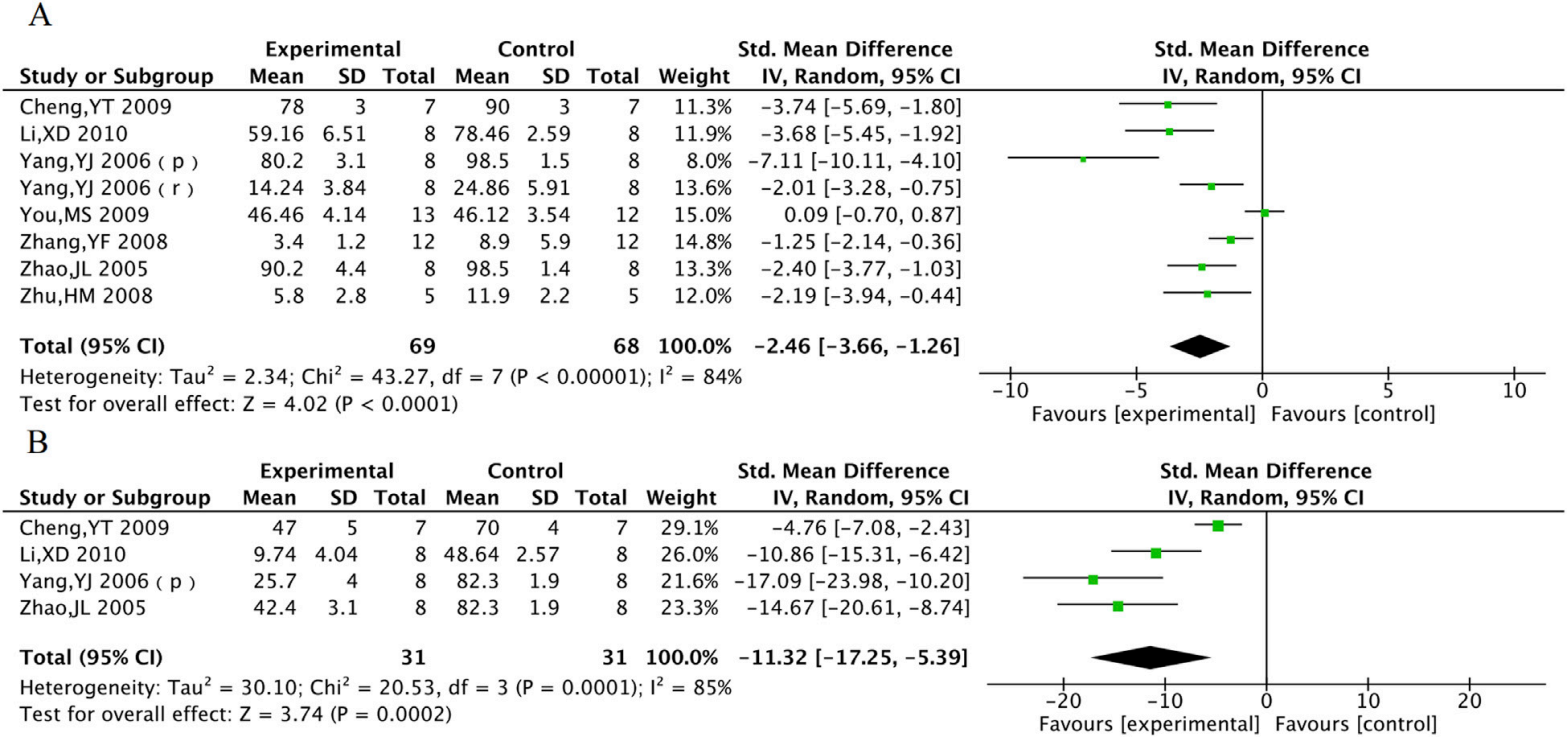
The meta-analysis result of myocardial infarction area **(A)** and no-reflow area **(B)** in animal models.

Myocardial infarction area was included in 8 animal studies involving 137 animals. No-reflow area was included in a total of 4 animal studies with 62 animals. The results of the meta-analysis showed that TXL group had significantly lower myocardial infarction area [SMD = −2.46, 95% CI (−3.66, −1.26), P < 0.001] and no-reflow area [SMD = −11.32, 95% CI (−17.25, −5.39), P < 0.001], compared with the control group (Figure 10).

#### 3.5.2 Cardiac function indicators

**FIGURE 11:**
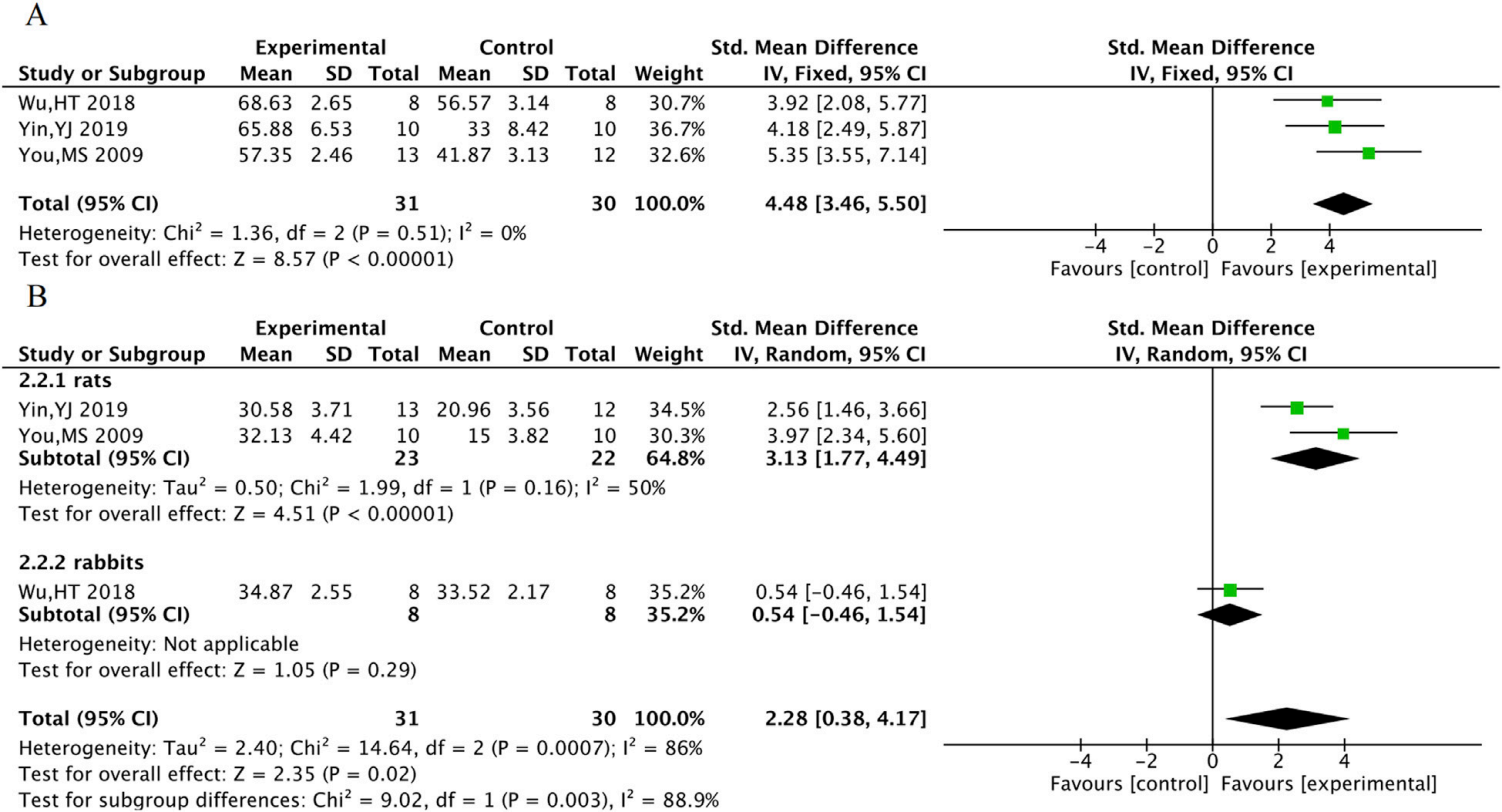
The meta-analysis result of cardiac function LVEF **(A)**, LVFS **(B)** in animals.

LVEF was included in a total of 3 animal studies including 61 animals, and LVFS was included in a total of 3 animal studies including 61 animals. The meta-analysis results indicate that TXL group had significantly higher LVEF [SMD = 4.48, 95% CI (3.46, 5.50), P < 0.001] and LVFS [SMD = 2.28, 95% CI (0.38, 4.17), P = 0.02]. In response to the high heterogeneity of LVFS, subgroup analyses were performed according to animal species. The heterogeneity within subgroups where the experimental animal was the rat was reduced (P = 0.16, I^2^ = 50%), suggesting stable outcomes in LVFS after TXL treatment in rats [SMDrats = 3.13, 95% CI (1.77, 4.49), P < 0.001] (Figure 11).

#### 3.5.3 cNOS and ET

**FIGURE 12:**
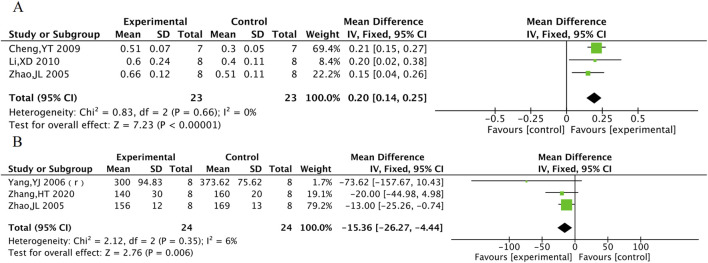
The meta-analysis result of cNOS **(A)**, ET **(B)** in animals.

cNOS was evaluated in 3 animal studies with a total of 46 animals, and ET was evaluated in 3 animal studies with 48 animals. The results of the meta-analysis showed that TXL group had a significant increase in cNOS activity [MD = 0.2, 95% CI (0.14, 0.25), P < 0.001] and a significant decrease in ET levels [MD = −15.36, 95% CI (−26.27, −4.44), P = 0.006] compared to the control group (Figure 12).

### 3.6 Sensitivity analysis

**FIGURE 13:**
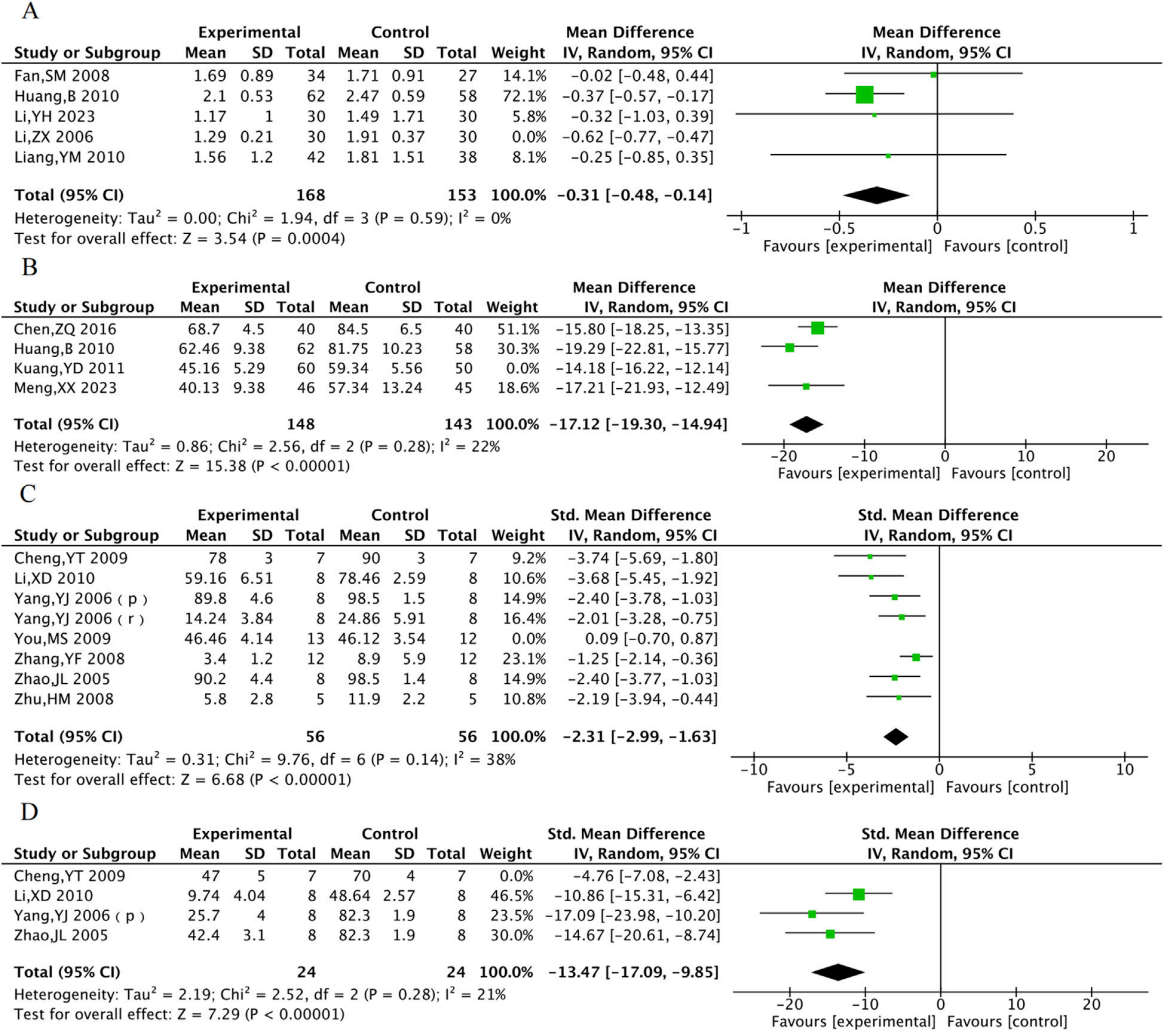
Sensitivity analysis of TG **(A)**, ET-1 **(B)**, Myocardial infarction area **(C)** and no-reflow area **(D)**.

We performed sensitivity analyses for outcomes with significant heterogeneity to assess the impact of individual trials on the combined results. In clinical trials, the heterogeneity in TG levels was resolved after excluding one study (Li, ZX 2006) that exclusively enrolled patients with acute anterior wall myocardial infarction, while the heterogeneity in ET-1 was resolved after excluding another study (Kuang, YD 2011) in which patients did not undergo PCI. The differences remained statistically significant in both cases (Figures 13A,B). Although significant heterogeneity persisted in the results for LVEF, CRP, and hs-CRP, the direction of effects remained consistent, indicating that the main conclusions are robust to methodological variations among the included studies. However, the observed heterogeneity may also be associated with publication bias, which will be evaluated in the following subsection. In animal studies, for myocardial infarction size, exclusion of one study (You, MS 2009) that used a staining method without Triphenyl tetrazolium Chloride led to a reduction in heterogeneity from 85% to 70%. Furthermore, the high-dose group data from another study (Yang, YJ 2006) was identified as an obvious outlier. Replacing this dataset with the low-dose group data from the same study (which used a dose comparable to other studies) further reduced heterogeneity to 38% (Figure 13C). These findings suggest that the high heterogeneity in myocardial infarction size is related to differences in measurement methods and drug dosage. Additionally, heterogeneity in the no-reflow area was eliminated after excluding one study (Cheng, YT 2009) that employed a different staining methodology (Figure 13D).

### 3.7 Publication bias

For the seven outcomes that each included more than 10 studies, publication bias was assessed using funnel plots (Supplementary Figure 1), Egger’s test, and trim-and-fill analysis (Table 5). For cardiovascular mortality, the result of Egger’s test showed an adjusted P = 0.8080, Z = −0.243, with a 95% CI of −0.7442 to −0.0103. For myocardial reinfarction, the result showed an adjusted P = 0.8048, Z = −0.2471, and a 95% CI of −1.1897 to −0.1437. For heart failure, the result showed Z = −0.5560, an adjusted P = 0.5782, and a 95% CI of −0.6774 to 0.2344. For angina pectoris, the result showed an adjusted P = 0.8281, Z = −0.2171, and a 95% CI of −1.5975 to 0.1050. For hs-CRP, the result showed tau = 4.9283, an adjusted P = 0.8759, and a 95% CI of −0.6626 to 4.1742. For CRP, the result showed tau = 4.5925, an adjusted P = 0.3567, and a 95% CI of −9.4550 to 9.8637. After trim-and-fill adjustment, the Egger’s test P-values for all outcomes exceeded 0.05, indicating a reduction in potential publication bias and improved reliability of the results. Notably, for left ventricular ejection fraction (LVEF), the trim-and-fill method indicated that no imputed studies were needed. The result remained consistent before and after adjustment (P = 0.0084), demonstrating robustness against publication bias. The funnel plots after trim-and-fill are provided in the Supplementary Material.

**TABLE 5:** Egger’s tests and Trim and Fill.

Outcome	Egger’s test, P value	Trim-and-Fill: imputed studies (side)	Adjusted, P value	95% CI	Tau/Z value	Notes
Cardiovascular mortality	0.2340	5 (R)	0.8080	(−0.7442, −0.0103)	−0.243	Potential bias noted
Incidence of myocardial reinfarction	0.4628	2 (R)	0.8048	(−1.1897, −0.1437)	−0.2471	Potential bias noted
Incidence of heart failure	0.0270	6 (R)	0.5782	(−0.6774, 0.2344)	−0.5560	Potential bias noted
Incidence of angina pectoris	0.3978	3 (R)	0.8281	(−1.5975, 0.1050)	−0.2171	Potential bias noted
LVEF	0.0084	0 (L)	0.0084	(5.5813, 1.9898)	2.3506	Robust
hs−CRP	0.0045	5 (R)	0.8759	(−0.6626, 4.1742)	4.9283	Potential bias noted
CRP	0.0145	2 (R)	0.3567	(−9.4550, 9.8637)	4.5925	Potential bias noted

Note: The numbers are all rounded to four decimal places.

### 3.8 Summary of findings and certainty of evidence

We assessed the certainty of evidence for all primary outcomes using the GRADE approach. The evidence for the critical outcomes (MACE) was rated as moderate certainty. Among the important outcomes, the certainty of evidence was assessed as low for LVEF, TC, triglycerides, and endothelin-1; moderate for LDL-C and HDL-C; and very low for hs-CRP and CRP.

The downgrading of evidence was primarily due to risk of bias (e.g., unclear allocation and/or blinding), imprecision (wide confidence intervals), and considerable heterogeneity observed in some outcomes. Although the certainty of evidence is limited for several outcomes, the consistent direction of effect across critical outcomes suggests that the main findings are reasonably robust. Detailed reasons for downgrading and full assessments are provided in the Summary of Findings table of Supplementary Material.

## 4 Discussion

In traditional medicine, AMI is categorized as “true heart attack.” Its pathogenesis is characterized by the obstruction of chest yang due to pathogenic factors, leading to functional impairment of chest yang and blockage of the heart vessels. The most common TCM pattern identified is qi deficiency combined with blood stasis (Doctor Society of Integrative Medicine and Chinese Medical Doctor Association, 2018). This condition often arises from aging or chronic illness that depletes qi and blood. Qi deficiency results in weakened blood circulation, which over time leads to blood stasis and obstruction of the heart vessels. Additionally, deficiency in the vessel network affects blood supply to the meridians, contributing to the formation of internal wind, which further exacerbates blockage of the heart vessels through disruption of qi flow.

TXL is a compound formulation of traditional Chinese medicine. Its founder, Academician Wu Yiling, based on the theory of veins and collaterals, added insect drugs with Chinese medicine characteristics of opening up the collaterals and activating the blood, such as leeches, scorpions, centipedes, so that TXL has the effect of activating the blood and removing blood stasis, eliminating the wind and relieving the pain, and removing the toxins and dispersing the lumps and knots (Han and Zhang, 2004; Wen et al., 2004; Xiao et al., 2002). TXL combines tonic and blood activating natural medicines. The tonic botanical drugs primarily include Ginseng and Sour Jujube Seed, which can nourish the heart, calm the mind, strengthen the lungs, strengthen the spleen, nourish the yin to reduce sweating. The blood-activating natural medicines are Sandalwood, Frankincense, Leech, Scorpion, and Centipede, which work to improve blood circulation, remove blood stasis, regulate qi to clear meridian obstructions while also dispersing wind and relieving pain. The two types of drugs are mutually reinforcing, and this dual action of activating blood flow and unblocking meridians complements the tonic effects, resulting in improved qi and blood flow, invigorated chest yang, and unobstructed heart meridians, leading to pain relief. Consequently, TXL improves both the clinical symptoms and prognosis of AMI by promoting blood circulation, removing blood congestion and unblocking the meridians.

In this study, we comprehensively searched currently published clinical trials in English and Chinese to describe the effects and safety of TXL. Compared with previous meta-analyses of TXL for acute myocardial infarction (AMI) (Li et al., 2018; Zhao et al., 2024; Chen et al., 2025), this study represents a substantial advance in both methodology and mechanistic exploration. Firstly, by implementing a more comprehensive literature search, this analysis incorporated newer and larger clinical trials and innovatively integrated a meta-analysis of animal studies into the overall evidence framework. Secondly, a more refined categorization of outcome measures-encompassing MACE, cardiac function, blood lipid profiles, and inflammatory markers-allowed for a more holistic quantification of Tongxinluo’s clinical benefits. Most importantly, by integrating clinical outcome data with evidence from mechanistic animal studies, this work seeks to explore the underlying multi-target mechanisms of Tongxinluo. This approach aims to extend the traditional observations of clinical efficacy toward mechanistic explanations, thereby enhancing the depth and translational value of our conclusions. Combining the meta-analyses results with currently published studies on TXL, the potential mechanisms of TXL in AMI may be summarized as follows.

### 4.1 Inhibit endothelial cell apoptosis and protect vascular endothelium

NO and ET are two major vasoactive substances secreted by vascular endothelial cells. Under physiological conditions, NO exerts protective effects by regulating vascular tone, inhibiting the activation, aggregation, and adhesion of neutrophils and platelets to the endothelium, whereas ET is a potent vasoconstrictor. Studies indicate that reduced NO release and increased ET release following ischemia-reperfusion are key factors in endothelial injury after myocardial reperfusion, and an increase in cNOS activity may promote NO release. Meta-analysis showed that TXL significantly increased cNOS activity and decreased plasma ET levels after reperfusion compared with control group, suggesting a protective effect on endothelial cell function in AMI. The underlying mechanism may involve TXL-mediated activation of the protein kinase A (PKA) pathway (Li et al., 2010) or the PI3K/Akt/HIF pathway (Liang et al., 2011), leading to phosphorylation of endothelial nitric oxide synthase (eNOS) and enhanced NO synthesis and endothelial function. Additionally, myocardial cells can interact with endothelial cells via exosomes, promoting eNOS production and NO release (Chen et al., 2020). TXL also protects endothelial cells from chondroitin-induced injury via the AMPK pathway and enhances their antioxidant capacity, supporting cardiovascular health (Zhang L. et al., 2008).

*In vitro* experiments demonstrated that TXL antagonized hypoxia-induced endothelial cell injury by modulating inflammation-associated cyclooxygenase-2, inducible nitric oxide synthase, and hypoxia-inducible factor-2α/vascular endothelial growth factor (VEGF) (Li et al., 2015). TXL protects cardiac microvascular endothelial cells (CMECs) from ischemia/reperfusion(I/R) injury via the MEK/ERK pathway, activating autophagy and protecting against I/R-induced apoptosis (Cui et al., 2014). Furthermore, TXL can reverse hypoxia-inhibited claudin-9 by upregulating H3K9ac in the claudin-9 gene promoter in endothelial cells, thereby strengthening intercellular tight junctions and protecting CMECs (Liu et al., 2016).

### 4.2 Suppresses inflammation and protect the myocardium

A major cause of structural and functional damage after AMI is the acute inflammatory response, which injures the myocardium, leading to myocardial cell death, fibrosis, and ventricular remodeling (Bekkers et al., 2010), and potentially heart failure (Li et al., 2017a). Meta-analysis results indicated that TXL can increase LVEF and LVFS after AMI, suggesting improved ventricular wall motion, attenuated ventricular remodeling, and enhanced cardiac function. This may be related to the fact that TXL reduces neutrophil infiltration in the infarct border zone.

Meta-analysis results indicated that TXL directly protected cardiomyocytes through multiple pathways. TXL treatment during I/R downregulated miR-128-3p in cardiomyocytes, increasing the phosphorylation and expression of p70s6k1, thereby inhibiting apoptosis via the miR-128-3p/p70s6k1 pathway and attenuating myocardial reperfusion injury (Chen et al., 2017). Another study showed that TXL reduced cardiomyocyte apoptosis by promoting AMPK phosphorylation and decreasing mTOR phosphorylation, activating autophagy during post-infarction cardiac ischemia (Li et al., 2017a). The resulting reduction in cardiomyocyte cell death is consistent with the meta-analysis findings of reduced infarct area and no-reflow area.

TXL also activates the PKA pathway to inhibit inflammation and edema, counteracting I/R-induced cardiac injury. It suppresses the expression of pro-inflammatory factors TNF-α and P-selectin, mitigates inflammatory cell death, and reduces the expression of aquaporin-4 to alleviate tissue, cellular, and mitochondrial edema caused by I/R (Li et al., 2013).

### 4.3 Promote angiogenesis

Studies have shown that TXL-induced angiogenesis is mediated through hypoxia-induced endothelial proliferation and tube formation (Zheng et al., 2015). Animal studies have shown that TXL promotes endothelial cell proliferation, migration and tube formation after infarction, potentially related to VEGF upregulation (Zhang, 2015). Proteomic studies have shown that angiopoietin-like protein 4 and VEGF specifically target endothelial cells, and TXL promotes angiogenesis by protecting these cytokines (Li et al., 2017b). Previous research demonstrated that TXL enhances angiogenesis in the ischemic heart, increased capillary density around infarct areas and improved blood perfusion in peri-infarct regions, which in turn repaired cardiac tissue (Li et al., 2017a; Wang et al., 2014), attenuated cardiac remodeling, and improved cardiac function, potentially through activation of signaling pathways such as Notchl/Jaggedl/VEGF, PI3K/AKT and ERK (Bai, 2014; Wang, 2016).

### 4.4 Lower blood lipids and stabilize plaque

**FIGURE 14:**
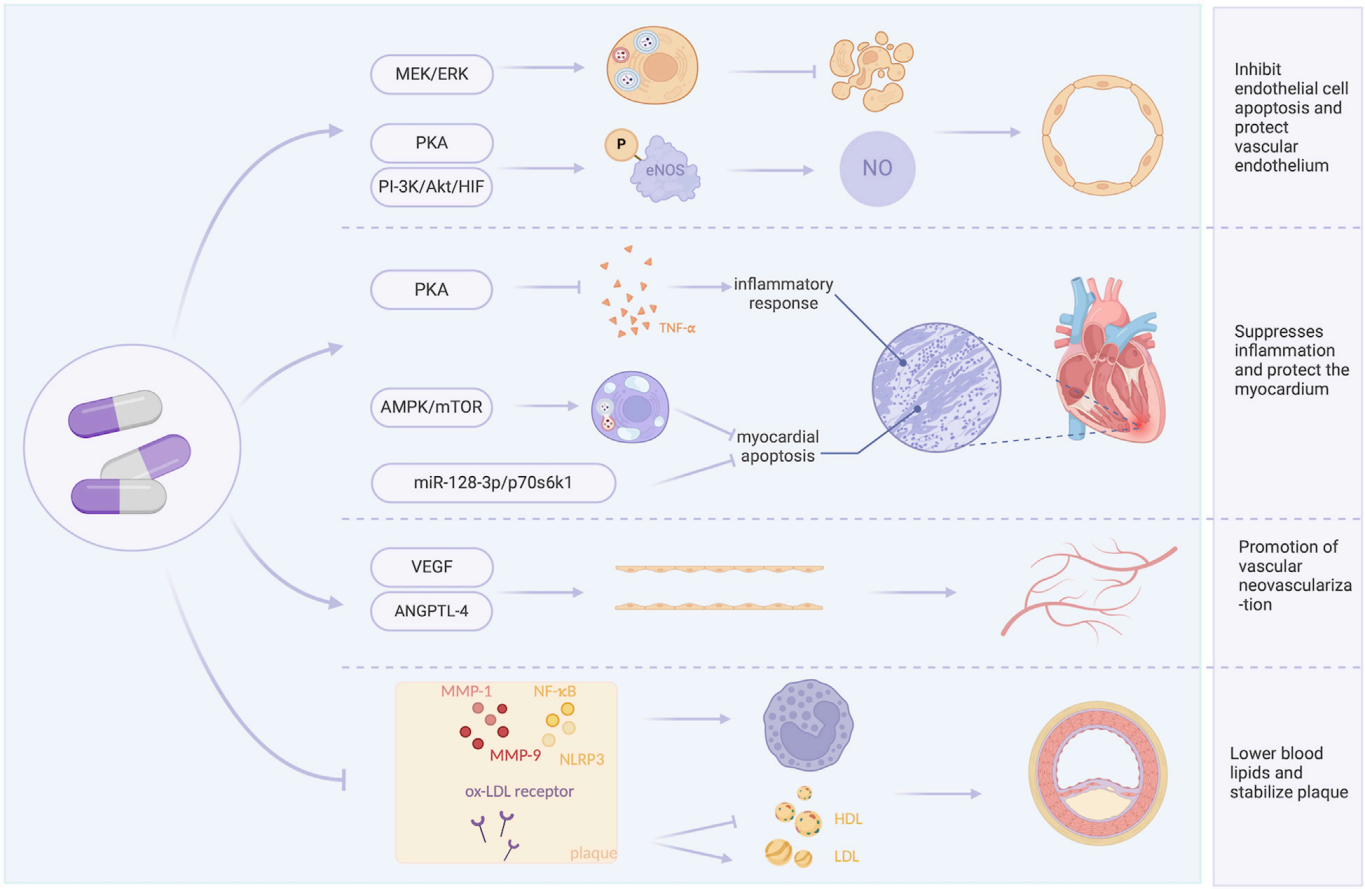
Potential mechanisms of TXL for the treatment of AMI.

Meta-analysis of clinical trial results showed that TXL can reduce LDL-C and increase HDL-C. Experimental evidence has shown that TXL reduced blood lipid levels dose-dependently, suppressed local and systemic inflammation, and exerted antioxidant effects, thereby increasing the stability of vulnerable plaques (Zhang et al., 2009). The CAPITAL study showed that adjuvant TXL treatment delayed the progression of mean carotid intima-media thickness, plaque area and vascular remodeling in patients with carotid atherosclerosis (Zhang et al., 2019). Anti-inflammatory effects are an important mechanism for plaque stabilization. Hs-CRP is a strong predictor of coronary events among inflammatory markers and is involved in processes such as plaque rupture. An animal study showed that TXL reduced serum levels of ICAM-1, VCAM-1 and hs-CRP in atherosclerotic mice, inhibited the expression of adhesion molecules in plaques and protected the endothelium from inflammatory infiltration, thereby stabilizing atherosclerotic plaques, which is consistent with our meta-analysis results (Liu et al., 2007). TXL may exert its anti-inflammatory effects by inhibiting the expression of oxidized low-density lipoprotein receptor 1, matrix metalloproteinase-1, matrix metalloproteinase-3, and nuclear factor-κB in plaques (Zhang L. et al., 2008). Another study found that the mechanism by which TXL improved plaque stability may involve promotion of gut flora metabolite trans-ferulic acid, which inhibited the macrophage NLRP3 inflammatory pathway (Qi et al., 2022). Gene chip analysis of TXL-treated atherosclerotic mice suggested that beyond inflammation and lipid metabolism, TXL may also stabilize plaques through hormonal, immune response, signaling, and cellular physiological functions (Ma et al., 2019). A schematic summary of the potential mechanisms of TXL in the treatment of AMI is provided in Figure 14.

### 4.5 Limitations of the study

First, as the majority of included patients were of Chinese origin, the generalizability of the results to European and American populations remains uncertain. Further clinical evaluations in other ethnic groups are warranted. Second, substantial clinical heterogeneity was observed across the included trials. Variations in patient risk profiles, infarction severity, TXL dosage, treatment duration, and intervention protocols may influence the pooled effect estimates of TXL for AMI. Third, the potential for publication bias must be acknowledged, as positive findings are more likely to be published, which may lead to an overestimation of the treatment effect. Fourth, most trials had short follow-up periods, limiting insight into the long-term clinical benefits of TXL. An ongoing real-world study (ITMCTR2100005609) is evaluating outcomes in the medium and long term; future meta-analyses should incorporate its findings to enhance the robustness of risk-benefit assessments in real-world settings. Fifth, the methodological quality of many included trials was suboptimal. Common issues included inadequate blinding, unclear allocation concealment, small sample sizes, and lack of *a priori* sample size calculations. Future clinical trials on traditional Chinese medicine should adhere to the CONSORT-TCM guidelines (Zheng et al., 2019) to improve reporting quality and transparency. Sixth, although TXL was generally associated only with mild gastrointestinal adverse events, evidence regarding its drug-drug interactions, long-term safety, and use in patients with hepatic or renal impairment remains scarce. Caution is advised, especially when co-administering with antithrombotic agents. Seventh, translational limitations exist between animal and human studies. Animal models of MI cannot fully replicate the complex pathophysiology of human AMI, which often involves comorbidities such as hyperlipidemia and type 2 diabetes. Consequently, mechanistic insights derived from animal studies may not wholly explain clinical outcomes. Eighth, the active metabolites of TXL and its precise pharmacologic mechanisms have not been fully elucidated. Further preclinical studies are needed to identify its key bioactive components and their modes of action.

## 5 Conclusion

This study presents the first comprehensive integration of evidence from both animal experiments and clinical trials regarding the use of TXL in the treatment of AMI. The results showed that TXL was associated with reduced myocardial reperfusion injury, a decreased incidence of myocardial no-reflow, and improved left ventricular systolic function, leading to significantly improved patient prognosis with an acceptable safety profile. Animal studies have shown that the mechanisms underlying TXL’s beneficial effects may involve the inhibition of apoptosis, suppression of inflammation, protection of cardiomyocytes and endothelial cells, promotion of angiogenesis, as well as synergistic lipid-lowering and plaque-stabilizing actions.

## Data Availability

The original contributions presented in the study are included in the article/Supplementary Material, further inquiries can be directed to the corresponding author.

## Supplementary material

SUPPLEMENTARY FIGURE 1 Funnel plot **(A)** Cardiovascular mortality. **(B)** Incidence of myocardial reinfarction. **(C)** Incidence of heart failure. **(D)** Incidence of angina pectoris. **(E)**. LVEF. **(F)** hs-CRP. **(G)** CRP.

## Abbreviations

TXL, Tongxinluo Capsule; AMI, acute myocardial infarction; PCI, percutaneous coronary intervention; MIRI, myocardial ischemia/reperfusion injury; NRP, no-reflow phenomenon; TCM, Traditional Chinese Medicine; RCTs, randomized controlled trials; LVEF, left ventricular ejection fraction; TC, total cholesterol; TG, triglycerides; HDL-C, high-density lipoprotein cholesterol; LDL-C, low-density lipoprotein cholesterol; hs-CRP, hypersensitive C-reactive protein; CRP, C-reactive protein; NO, nitric oxide; ET, endothelin; LVFS, left ventricular fractional shortening; cNOS, Constitutive nitric oxide synthase; SYRCLE, SYstematic Review Centre for Laboratory animal Experimentation; RR, Relative risk; MD, mean difference; SMD, standardized mean difference; eNOS, endothelial nitric oxide synthase; CMEC, cardiac microvascular endothelial cells; VEGF, vascular endothelial growth factor; I/R, ischemia/reperfusion.
