# Supplementary material for: Tongxinluo capsule for acute myocardial infarction: a systematic review and meta-analysis
Source: Front Pharmacol. 2025 Oct 22;16:1632809. doi: 10.3389/fphar.2025.1632809 (PMC12587000; doi:10.3389/fphar.2025.1632809)
Supplement: Supplementary file 1 [file Supplementaryfile1.docx]

Supplementary Material

# The search strategies of Tongxinluo capsule for Myocardial Infarction

| **Databases** | **search strategies** | **Hit Counts** |
| --- | --- | --- |
| **Pubmed (https://pubmed.ncbi.nlm.nih.gov/advanced/)** | #1: All Fields= “Tongxinluo capsule” OR “Tongxinluo” OR “tongxinluo capsule”  #2: All Fields= “Myocardial Infarction” OR “Infarction, Myocardial” OR “Infarctions, Myocardial” OR “Myocardial Infarctions” OR “Cardiovascular Stroke” OR “Cardiovascular Strokes” OR “Stroke, Cardiovascular” OR “Strokes, Cardiovascular” OR “Myocardial Infarct” OR “Infarct, Myocardial” OR “Infarcts, Myocardial” OR “Myocardial Infarcts” OR “Heart Attack” OR “Heart Attacks”  #3: #1 and #2 | 27 |
| **Web of science**  **(https://www.webofscience.com/)** | #1: TS= “Tongxinluo capsule” OR “Tongxinluo” OR “tongxinluo capsule”  #2: TS= “Myocardial Infarction” OR “Infarction, Myocardial” OR “Infarctions, Myocardial” OR “Myocardial Infarctions” OR “Cardiovascular Stroke” OR “Cardiovascular Strokes” OR “Stroke, Cardiovascular” OR “Strokes, Cardiovascular” OR “Myocardial Infarct” OR “Infarct, Myocardial” OR “Infarcts, Myocardial” OR “Myocardial Infarcts” OR “Heart Attack” OR “Heart Attacks”  #3: #1 and #2 | 52 |
| **Embase**  **(https://www.embase.com/#advancedSearch)** | #1: Broad search= ‘Tongxinluo capsule’ OR ‘Tongxinluo’ OR ‘tongxinluo capsule’  #2: Broad search= ‘Myocardial Infarction’ OR ‘Infarction, Myocardial’ OR ‘Infarctions, Myocardial’ OR ‘Myocardial Infarctions’ OR ‘Cardiovascular Stroke’ OR ‘Cardiovascular Strokes’ OR ‘Stroke, Cardiovascular’ OR ‘Strokes, Cardiovascular’ OR ‘Myocardial Infarct’ OR ‘Infarct, Myocardial’ OR ‘Infarcts, Myocardial’ OR ‘Myocardial Infarcts’ OR ‘Heart Attack’ OR ‘Heart Attacks’  #3: #1 and #2 | 108 |
| **The Cochrane Library**  **(https://www.cochranelibrary.com/advanced-search)** | #1: “Tongxinluo capsule” OR “Tongxinluo ” OR “tongxinluo capsule”  #2: “Myocardial Infarction” OR “Infarction, Myocardial” OR “Infarctions, Myocardial” OR “Myocardial Infarctions” OR “Cardiovascular Stroke” OR “Cardiovascular Strokes” OR “Stroke, Cardiovascular” OR “Strokes, Cardiovascular” OR “Myocardial Infarct” OR “Infarct, Myocardial” OR “Infarcts, Myocardial” OR “Myocardial Infarcts” OR “Heart Attack” OR “Heart Attacks”  #3: #1 and #2 | 20 |
| **CNKI**  **(https://www.cnki.net/)** | ((FT=('通心络' + '通心络胶囊') AND FT=('急性心肌梗死' + '急性心梗')) AND ((SU=('通心络'+ '通心络胶囊') AND SU=('急性心肌梗死' + '急性心梗')) AND ((TKA=('通心络' + '通心络胶囊') AND TKA=('急性心肌梗死' + '急性心梗')) | 151 |
| **WanFang**  **(https://w.wanfangdata.com.cn/)** | 全部: ("通心络" or "通心络胶囊") and 全部: ("急性心肌梗死" or "急性心梗") | 295 |
| **VIP**  **(http://www.cqvip.com/)** | U=(通心络 OR 通心络胶囊) and U=(急性心肌梗死 OR 急性心梗) | 359 |
| **SinoMed**  **(http://www.sinomed.ac.cn/cross/advancedSearch.jsp)** | ( "通心络"[全部字段:智能] OR "通心络胶囊"[全部字段:智能]) AND ( "急性心肌梗死"[全部字段:智能] OR "急性心梗"[全部字段:智能]) | 161 |
| **the Chinese Clinical Trial Registry (https://www.chictr.org.cn/searchproj.html)** | In the "Intervention Measures" search box, enter "Tongxinluo Capsules" | 7 |
| **The World Health Organization International Clinical Trials Registry Platform**  **(https://trialsearch.who.int/AdvSearch.)** | #1 (Title): Tongxinluo capsule OR Tongxinluo OR tongxinluo capsule  #2 (Condition): Myocardial Infarction OR Infarction, Myocardial” OR “Infarctions, Myocardial OR Myocardial Infarctions OR Cardiovascular Stroke OR Cardiovascular Strokes OR Stroke, Cardiovascular OR Strokes, Cardiovascular OR Myocardial Infarct OR Infarct, Myocardial OR Infarcts, Myocardial OR Myocardial Infarcts OR Heart Attack OR Heart Attacks  #3: #1 and #2 | 1 |
| **ClinicalTrials.gov**  **(https://trialsearch.who.int/AdvSearch.aspx)** | #1 (Intervention/trail): Tongxinluo capsule OR Tongxinluo OR tongxinluo capsule  #2 (Title/Acronym): Myocardial Infarction OR Infarction, Myocardial” OR “Infarctions, Myocardial OR Myocardial Infarctions OR Cardiovascular Stroke OR Cardiovascular Strokes OR Stroke, Cardiovascular OR Strokes, Cardiovascular OR Myocardial Infarct OR Infarct, Myocardial OR Infarcts, Myocardial OR Myocardial Infarcts OR Heart Attack OR Heart Attacks  #3: #1 and #2 | 0 |
| **International Traditional Medicine Clinical Trial Registry** | In the "Public TItle " search box, enter "Tongxinluo" | 1 |

# Table 1: Basic Characteristics of 54 Clinical Studies. Note: T: trail group; C: control group; AMI: acute myocardial infarction; STEMI: ST-segment elevation myocardial infarction; TXL: Tongxinluo capsule; GDMT: guideline-directed medical therapy; NT: not mentioned. ①: All-cause mortality, cardiovascular mortality, incidence of myocardial reinfarction; ②: Incidence of repeat revascularization, heart failure, angina pectoris, arrhythmias; ③LVEF; ④Lipid profiles; ⑤Inflammatory markers; ⑥Adverse drug reactions.

| **Study ID** | **Gander**  **(male/female)** | | **age** | | | **Sample size (T/C)** | **Disease** | **T** | **C** | **Usage and dosage, duration** | **Outcomes** |
| --- | --- | --- | --- | --- | --- | --- | --- | --- | --- | --- | --- |
|  | **T** | **C** | **T** | **C** | |  |  |  |  |  |  |
| Yang, YJ 2023 | 1456/433 | 1448/440 | 61.4±12.1 | 61.5±12.1 | | 3777 (1889/1888) | STEMI | TXL plus GDMT | GDMT plus  Placebo | Pre-PCI: 8 capsules, tid; post-PCI: 4 capsules, tid;12months | ①②⑥ |
| Meng, XX 2023 | 24/22 | 26/19 | 52.06±9.69 | 51.28±10.03 | | 91 (46/45) | AMI, post-PCI | TXL plus GDMT | GDMT | 3 capsules, tid;12 weeks | ①②⑤ |
| Ren, FB 2023 | 14/31 | 20/25 | 56.27**±**6.68 | 56.31**±**6.34 | | 90 (45/45) | AMI | TXL plus GDMT | GDMT | 5 capsules, tid;2 weeks | ①②③⑤ |
| Li, YH 2023 | 16/14 | 15/15 | 61.05±14.85 | 60.88±15. 09 | | 60 (30/30) | AMI | TXL plus GDMT | GDMT | 4 capsules, tid;8 weeks | ①②④ |
| Zhou, CJ 2022 | 21/13 | 20/14 | 49.82 ± 6.18 | 50.09±5.86 | | 68 (34/34) | AMI, perioperative period | TXL plus GDMT | GDMT | 2-4 capsules, tid;4 weeks | ②⑤ |
| Mai, QX 2022 | 21/14 | 22/13 | 59.47±8.65 | 59.59±8.36 | | 70 (35/35) | AMI | TXL plus GDMT | GDMT | 2 capsules, tid;1 week | ③⑤ |
| Yu, YM 2021 | 29/17 | 28/18 | 58.5±5.0 | 57.9±4.8 | | 92 (46/46) | AMI, post-PCI | TXL plus GDMT | GDMT | 3 capsules, tid;12 weeks | ③⑥ |
| Shi, ML 2021 | 23/19 | 22/20 | 53.12±8.95 | 52.36±10.48 | | 84 (42/42) | AMI | TXL plus GDMT | GDMT | 4 capsules, tid;15 days | ③ |
| Huang, J 2021 | 26/15 | 26/16 | 65.24±7.11 | 65.52±7.25 | | 83 (41/42) | AMI | TXL plus GDMT | GDMT | 5 capsules, tid;8 weeks | ③⑤⑥ |
| Hong, ZZ 2021 | 30/27 | 28/29 | 63.05±5.72 | 62.58±5.65 | | 114 (57/57) | AMI, post-PCI | TXL plus GDMT | GDMT | 4 capsules, tid;12 weeks | ③⑥ |
| Wang, WW 2021 | 31/19 | 33/17 | 57.23±8.92 | 56.70±9.42 | | 100 (50/50) | AMI, post-PCI | TXL plus GDMT | GDMT | 4 capsules, tid;4 weeks | ③⑤ |
| Jia, HL 2021 | 22/23 | 25/20 | 45.39±7.02 | 44.82±6.73 | | 90 (45/45) | AMI | TXL plus GDMT | GDMT | 2 capsules, tid;4 weeks | ③⑥ |
| Shen, Q 2021 | 29/21 | 27/23 | 59.14±5.33 | 59.23±5.41 | | 100 (50/50) | AMI | TXL plus GDMT | GDMT | 5 capsules, tid;2 weeks | ③⑤⑥ |
| Yu, ZL 2021 | 31/29 | 36/24 | 55.98±10.04 | 57.65±9.76 | | 120 (60/60) | AMI, post-PCI | TXL plus GDMT | GDMT | 2-4 capsules, tid;8 weeks | ⑥ |
| Zhao, XP 2021 | 39/14 | 38/15 | 51-64 | 50-62 | | 106 (53/53) | AMI | TXL plus GDMT | GDMT | 2 capsules, tid;2 weeks | ⑥ |
| Zhao, YY 2020 | 40/32 | | 61.36±12.19 | | | 72 (38/34) | AMI, post-PCI | TXL plus GDMT | GDMT | Post-PCI: 8 capsules; post-PCI: 4 capsules, tid;4 weeks | ①③⑥ |
| Ge, ZQ 2020 | 35/25 | 38/22 | 42.7±5.2 | | 42.2±5.7 | 120 (60/60) | AMI, post-PCI | TXL plus GDMT | GDMT | 4 capsules, tid;24 weeks | ①②⑤ |
| Liu, H 2020 | 54/53 | 58/49 | 59.6±18.5 | | 57.1±15.3 | 214 (107/107) | AMI, post-PCI | TXL plus GDMT | GDMT | 3 capsules, tid;12 weeks | ③ |
| Ding, N 2020 | 28/15 | 25/18 | 56.11±6.23 | | 56.37±6.49 | 86 (43/43) | AMI | TXL plus GDMT | GDMT | 4 capsules, tid;2 weeks | ③⑤ |
| Zhou, LY 2019 | 29/18 | 27/20 | 56.87±6.59 | | 57.42±7.23 | 94 (47/47) | AMI | TXL plus GDMT | GDMT | 4 capsules, tid;4 weeks | ③⑤ |
| Chen, XY 2019 | 29/23 | 30/22 | 66.32±3.28 | | 67.82±3.74 | 104 (52/52) | AMI | TXL plus GDMT | GDMT | 4 capsules, tid;12 weeks | ③ |
| Xu, WW 2019 | 16/23 | 17/22 | 71.88±5.98 | | 70.56±6.48 | 78 (39/39) | AMI, post-PCI | TXL plus GDMT | GDMT | 2-4 capsules, tid;4 weeks | ② |
| Wang, CL 2018 | 30/16 | 28/18 | 52.51±12.66 | | 51.16±11.54 | 92 (46/46) | AMI | TXL plus GDMT | GDMT | 4 capsules, tid;12 weeks | ③⑤ |
| Zhou, S 2018 | 29/18 | 27/20 | 56.94 ± 4.61 | | 57.38±5.13 | 94 (47/47) | AMI | TXL plus GDMT | GDMT | 5 capsules, tid;2 weeks | ③⑤ |
| Peng, ZP 2017 | 43/42 | 45/40 | 61.3±3.8 | | 62.8 ± 3.5 | 170 (85/85) | AMI,  Post-PCI | TXL plus GDMT | GDMT | 4 capsules, tid;1 week | ①② |
| Wang, YL 2016 | 19/11 | 18/12 | 58±7 | | 58±6 | 60 (30/30) | AMI, post-PCI | TXL plus GDMT | GDMT | 4 capsules, tid;48 weeks | ③⑤ |
| Chen, ZQ 2016 | 28/12 | 26/14 | 60.5±14.6 | | 61.7±13.6 | 80 (40/40) | AMI, post-PCI | TXL plus GDMT | GDMT | 4 capsules, tid;1 week | ①②③⑤ |
| Tian, ZT 2014 | 22/8 | 19/11 | 54.9±10.4 | | 54.5±9.8 | 60 (30/30) | AMI, post-PCI | TXL plus GDMT | GDMT | 4 capsules, tid;12 weeks | ①②③⑤ |
| Dong, SQ 2012 | 45/18 | | 40-77 | | | 63 (35/28) | AMI, post-PCI | TXL plus GDMT | GDMT | 4 capsules, tid;48 weeks | ①⑤ |
| Wang, HZ 2012 | 38/24 | | 71.60±4.20 | | | 62 (31/31) | AMI | TXL plus GDMT | GDMT | 2 capsules, tid;20 days | ①②③ |
| Yang, W 2012 | 17/13 | 18/11 | 64±11 | 66±12 | | 59 (30/29) | AMI, post-PCI | TXL plus GDMT | GDMT | 3 capsules, tid;12 weeks | ③ |
| Kuang, YD2011 | 75/35 | | 56.34±10.82 | | | 110 (60/50) | STEMI | TXL plus GDMT | GDMT | 3 capsules, tid;30 days | ③⑤ |
| You, MS 2011 | 83/8 | | 54±5.6 | 47±5.2 | | 91 (45/46) | AMI | TXL plus GDMT | GDMT | 4 capsules, tid;8 weeks | ③ |
| Liao, CL 2010 | 21/18 | 20/17 | 60.3±9.9 | 64.3±11.7 | | 76 (39/37) | AMI | TXL plus GDMT | GDMT | 3 capsules, tid;2 weeks | ③⑤ |
| Huang, B 2010 | 68/52 | | 58.3±12.6 | | | 120 (62/58) | AMI, post-PCI | TXL plus GDMT | GDMT | 4 capsules, tid;24 weeks | ①④⑤ |
| Liang, YM 2010 | 52/28 | | 40-70 | | | 80 (42/38) | AMI, post-PCI | TXL plus GDMT | GDMT | 2 capsules, tid;24 weeks | ①②④ |
| Yang, W 2009 | 22/8 | 25/4 | 58 | 56 | | 59 (30/29) | AMI, post-PCI | TXL plus GDMT | GDMT | 3 capsules, tid;12 weeks | ①②③ |
| Zhang, XP 2009 | 70/26 | 57/25 | 60±5 | 58±8 | | 178 (96/82) | AMI | TXL plus GDMT | GDMT | 3 capsules, tid;96 weeks | ①②⑥ |
| Zhao, QH 2009 | 40/10 | 35/13 | 59.6 | 60.3 | | 98 (50/48) | AMI | TXL plus GDMT | GDMT | 3 capsules, tid;48 weeks | ①② |
| Fan, SM 2008 | 50/11 | | 56.23±12.06 | | | 61 (34/27) | AMI, post-PCI | TXL plus GDMT | GDMT | NT; 4 weeks | ④ |
| Chen, W 2008 | 23/12 | 25/10 | 68±7 | 69±7 | | 70 (35/35) | AMI | TXL plus GDMT | GDMT | 3 capsules, tid; 6 weeks | ③ |
| Wang, G 2007 | 31/3 | 30/4 | 58.08±11.14 | 57.69±11.18 | | 68 (34/34) | AMI, post-PCI | TXL plus GDMT | GDMT | 4 capsules, tid;24 weeks | ③ |
| Chen, H 2007 | 32/38 | | 58±11 | | | 60 (30/30) | AMI, post-PCI | TXL plus GDMT | GDMT | 2-4 capsules, tid;8 weeks | ①②③④⑤ |
| Li, ZX 2006 | 21/9 | 20/10 | 48-73 | 49-75 | | 60 (30/30) | AMI | TXL plus GDMT | GDMT | 3 capsules, tid;3 weeks | ④⑥ |
| You, SJ 2005 | 52/8 | 40/12 | 57.08±11.04 | 58.74±11.24 | | 112 (60/52) | AMI | TXL plus GDMT | GDMT | 4 capsules, tid;24 weeks | ③ |
| Zhang, JW 2006 | 43/18 | | 64±9 | | | 61  (30/31) | AMI, post-PCI | TXL plus GDMT | GDMT | 3 capsules, bid;2 weeks | ⑤ |
| Shen, X 2024 | 35/28 | 40/23 | 53.09±12.33 | 52.35±11.42 | | 126(63/63) | STEMI, post-PCI | TXL plus GDMT | GDMT | 3 capsules, tid;4 weeks | ⑤ |
| Adili2024 | 23/17 | 20/20 | 60.45±4.58 | 59.56±5.17 | | 80(40/40) | AMI, post-PCI | TXL plus GDMT | GDMT | 2 capsules, tid;24 weeks | ①②③⑤ |
| Li, P 2024 | 53/44 | 56/41 | 55.87±8.46 | 55.83±8.42 | | 194(97/97) | AMI, post-PCI | TXL plus GDMT | GDMT | 2-4 capsules, tid;8 weeks | ①②③⑤ |
| Xiong, L 2024 | 22/18 | 23/17 | 71.86±8.15 | 71.49±8.32 | | 80(40/40) | AMI, post-PCI | TXL plus GDMT | GDMT | 4 capsules, tid;12 weeks | ①②③ |
| Xu, YG 2024 | 27/19 | 25/21 | 58. 55±6. 81 | 58. 42±6. 75 | | 92(46/46) | AMI, post-PCI | TXL plus GDMT | GDMT | 4 capsules, tid;4 weeks | ③⑤⑥ |
| Yan, DY 2024 | 20/13 | 19/14 | 0.34±5.28 | 59.63±5.14 | | 66(33/33) | AMI | TXL plus GDMT | GDMT | 5 capsules, tid;2 weeks | ⑥ |
| Zhang, XP 2024 | 29/24 | 28/25 | 62.82±5.98 | 63.25±5.77 | | 106(53/53) | AMI, post-PCI | TXL plus GDMT | GDMT | 4 capsules, tid;4 weeks | ③⑤ |
| Yan, S 2024 | 26/19 | 25/20 | 58.85±5.87 | 59.52±6.58 | | 90(45/45) | AMI, post-PCI | TXL plus GDMT | GDMT | 4 capsules, tid;1 year | ③⑤ |

# Egger’s tests. Note: (A) Cardiovascular mortality (B) Incidence of myocardial reinfarction (C) Incidence of heart failure (D) Incidence of angina pectoris (E) LVEF (F) hs-CRP (G) CRP


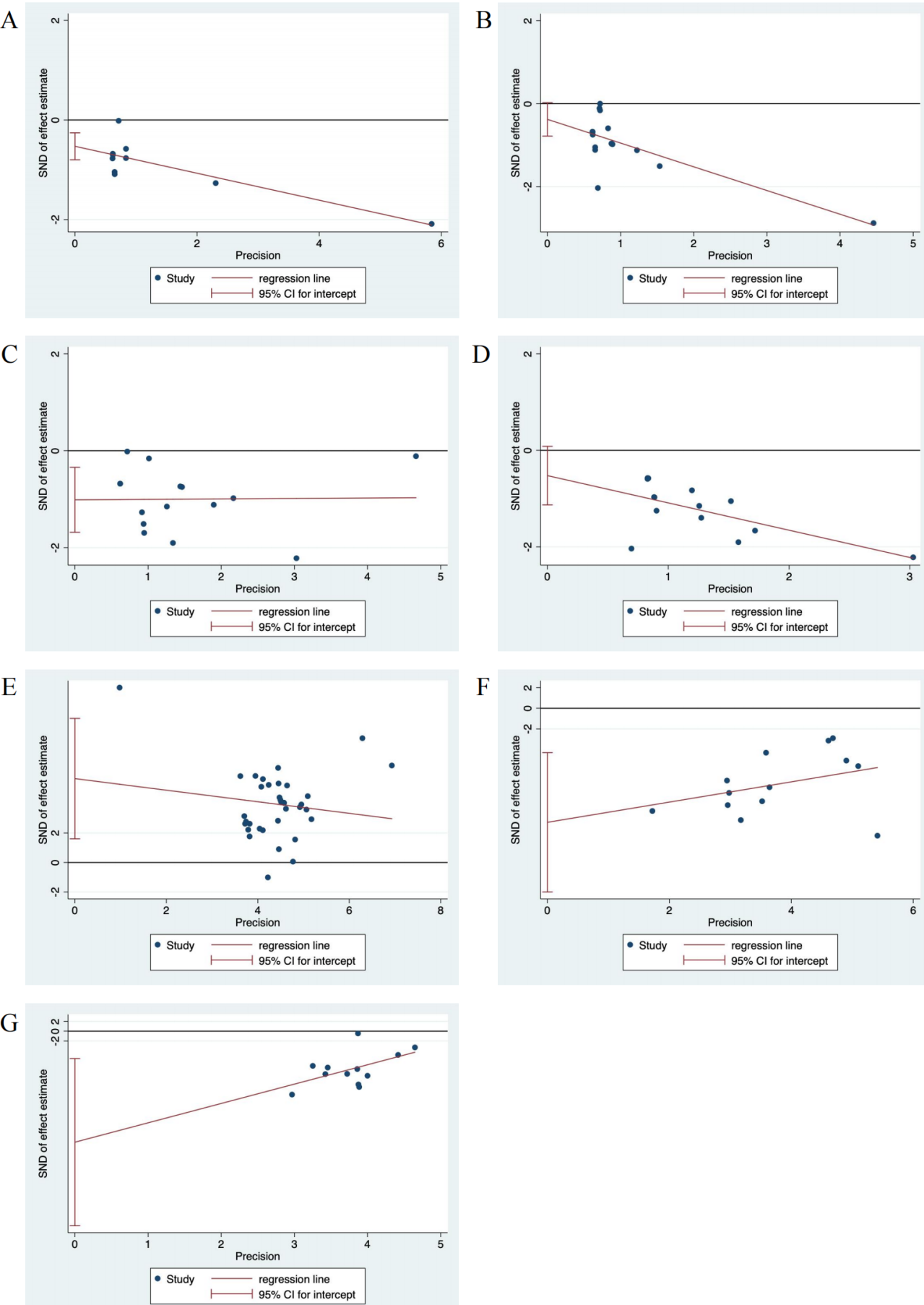


# The funnel plots after trim-and-fill.

#
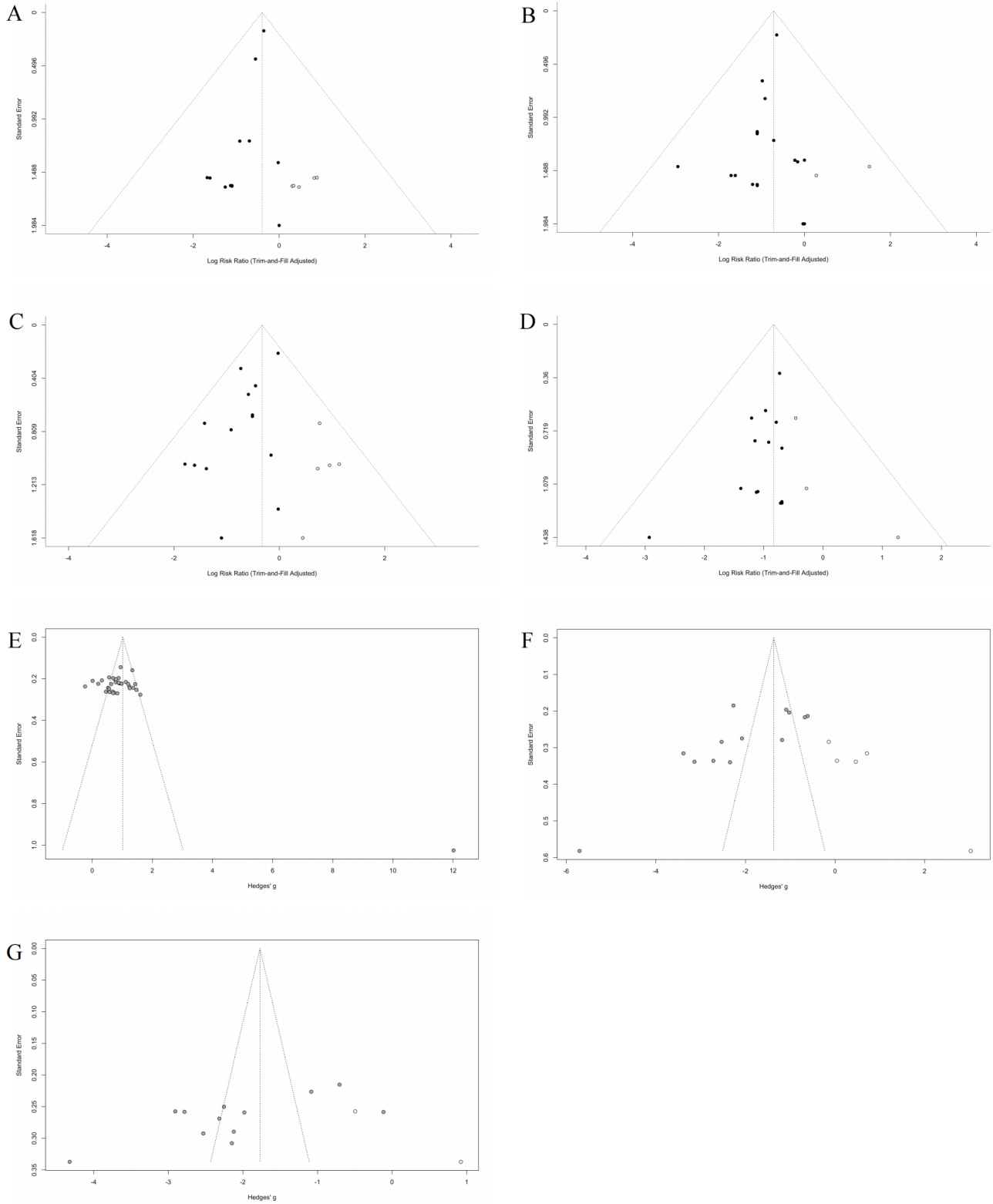


#
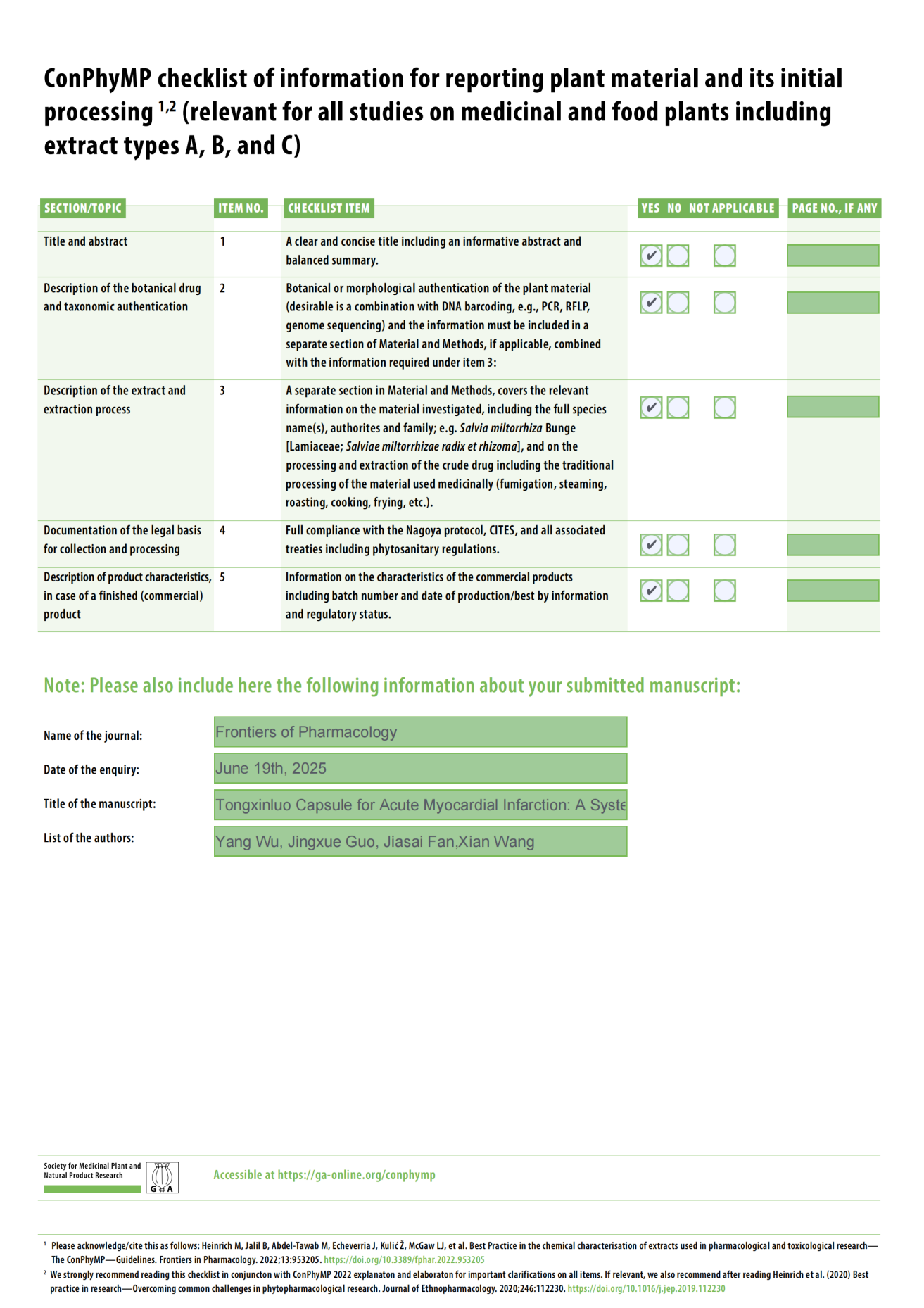
 A detailed report on the nomenclature standardization and verification of the botanical drugs in Tongxinluo Capsule was generated using the ConPhyMP (Consensus for Chemical Analysis of Plant-based Medicinal Products) tool.


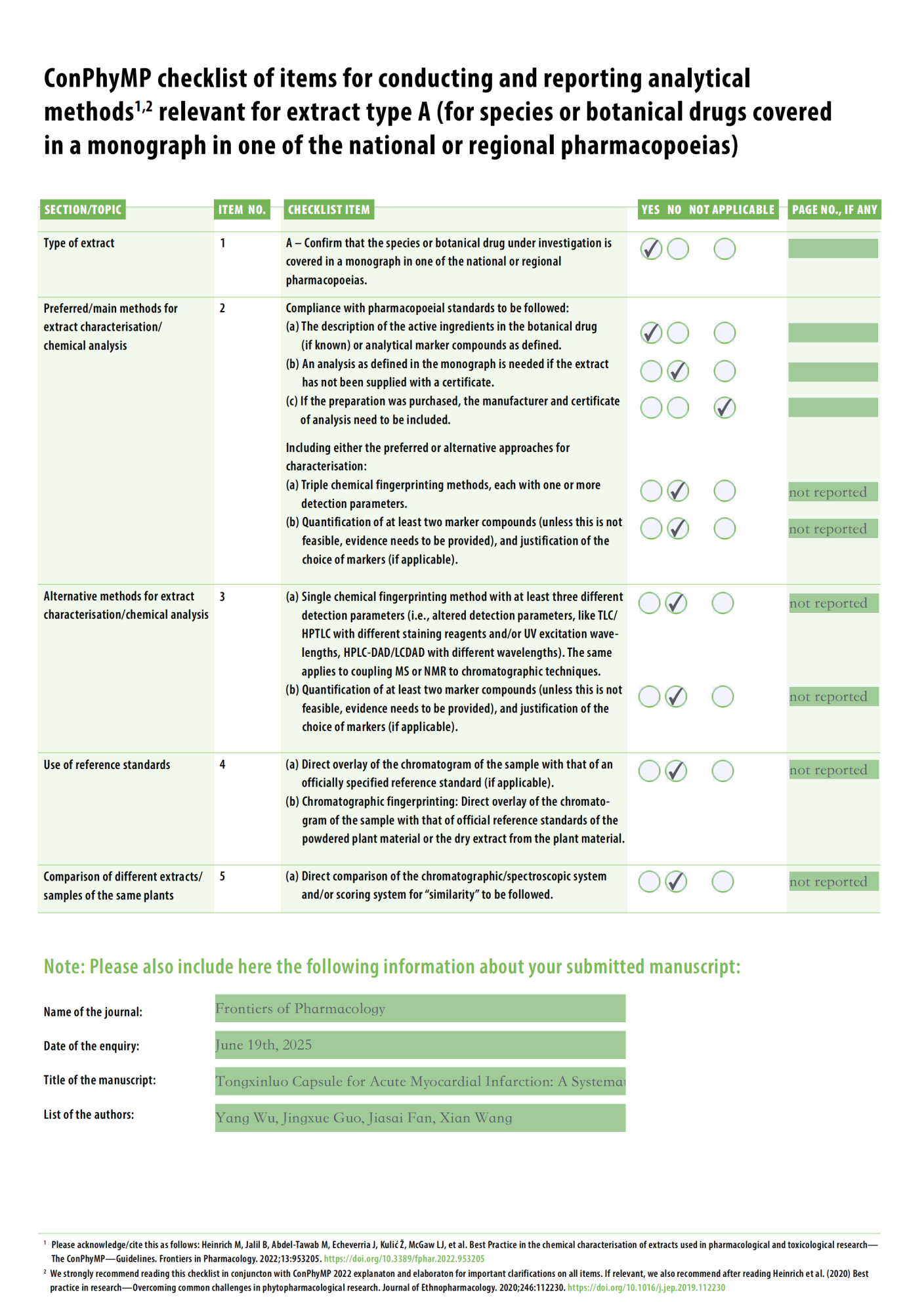


# Summary of Findings (SoF) Table: Tongxinluo Capsule versus Control for Acute Myocardial Infarction.

| **Certainty assessment** | | | | | | | **№ of patients** | | **Effect** | | **Certainty** | **Importance** |
| --- | --- | --- | --- | --- | --- | --- | --- | --- | --- | --- | --- | --- |
| **№ of studies** | **Study design** | **Risk of bias** | **Inconsistency** | **Indirectness** | **Imprecision** | **Other considerations** | **TXL** | **placebo** | **Relative (95% CI)** | **Absolute (95% CI)** |  |  |
| **all-cause mortality** | | | | | | | | | | | | |
| 4 | randomised trials | serious^a^ | not serious | not serious | not serious | none | 99/2054 (4.8%) | 131/1981 (6.6%) | **RR 0.75** (0.58 to 0.96) | **17 fewer per 1,000** (from 28 fewer to 3 fewer) | ⨁⨁⨁◯ Moderate^a^ | CRITICAL |
| **cardiovascular mortality** | | | | | | | | | | | | |
| 12 | randomised trials | serious^a^ | not serious | not serious | not serious | none | 65/2399 (2.7%) | 103/2370 (4.3%) | **RR 0.63** (0.47 to 0.85) | **16 fewer per 1,000** (from 23 fewer to 7 fewer) | ⨁⨁⨁◯ Moderate^a^ | CRITICAL |
| **incidence of myocardial reinfarction** | | | | | | | | | | | | |
| 18 | randomised trials | serious^a^ | not serious | not serious | not serious | none | 24/2721 (0.9%) | 65/2688 (2.4%) | **RR 0.39** (0.26 to 0.57) | **15 fewer per 1,000** (from 18 fewer to 10 fewer) | ⨁⨁⨁◯ Moderate^a^ | CRITICAL |
| **incidence of revascularization** | | | | | | | | | | | | |
| 8 | randomised trials | serious^a^ | not serious | not serious | not serious | none | 5/2272 (0.2%) | 19/2245 (0.8%) | **RR 0.28** (0.11 to 0.67) | **6 fewer per 1,000** (from 8 fewer to 3 fewer) | ⨁⨁⨁◯ Moderate^a^ | CRITICAL |
| **incidence of heart failure** | | | | | | | | | | | | |
| 15 | randomised trials | serious^a^ | not serious | not serious | not serious | none | 78/2528 (3.1%) | 122/2510 (4.9%) | **RR 0.64** (0.49 to 0.83) | **17 fewer per 1,000** (from 25 fewer to 8 fewer) | ⨁⨁⨁◯ Moderate^a^ | CRITICAL |
| **incidence of angina** | | | | | | | | | | | | |
| 14 | randomised trials | serious^a^ | not serious | not serious | not serious | none | 30/643 (4.7%) | 80/623 (12.8%) | **RR 0.37** (0.25 to 0.54) | **81 fewer per 1,000** (from 96 fewer to 59 fewer) | ⨁⨁⨁◯ Moderate^a^ | CRITICAL |
| **incidence of arrhythmia** | | | | | | | | | | | | |
| 7 | randomised trials | serious^a^ | not serious | not serious | not serious | none | 157/2169 (7.2%) | 216/2168 (10.0%) | **RR 0.73** (0.60 to 0.88) | **27 fewer per 1,000** (from 40 fewer to 12 fewer) | ⨁⨁⨁◯ Moderate^a^ | CRITICAL |
| **LVEF** | | | | | | | | | | | | |
| 35 | randomised trials | serious^a^ | serious^b^ | not serious | not serious | none | 1606 | 1583 | - | MD **4.61 higher** (3.92 higher to 5.29 higher) | ⨁⨁◯◯ Low^a,b^ | IMPORTANT |
| **TC** | | | | | | | | | | | | |
| 5 | randomised trials | serious^a^ | serious^b^ | not serious | not serious | none | 186 | 175 | - | MD **0.93 lower** (1.26 lower to 0.6 lower) | ⨁⨁◯◯ Low^a,b^ | IMPORTANT |
| **TG** | | | | | | | | | | | | |
| 5 | randomised trials | serious^a^ | serious^b^ | not serious | not serious | none | 198 | 183 | - | MD **0.39 lower** (0.61 lower to 0.17 lower) | ⨁⨁◯◯ Low^a,b^ | IMPORTANT |
| **HDL-C** | | | | | | | | | | | | |
| 3 | randomised trials | serious^a^ | not serious | not serious | not serious | none | 102 | 98 | - | MD **0.07 higher** (0.01 higher to 0.13 higher) | ⨁⨁⨁◯ Moderate^a^ | IMPORTANT |
| **LDL-C** | | | | | | | | | | | | |
| 3 | randomised trials | serious^a^ | not serious | not serious | not serious | none | 102 | 98 | - | MD **0.58 lower** (0.7 lower to 0.47 lower) | ⨁⨁⨁◯ Moderate^a^ | IMPORTANT |
| **hs-CRP** | | | | | | | | | | | | |
| 13 | randomised trials | serious^a^ | very serious^b^ | not serious | not serious | none | 617 | 597 | - | MD **4.88 lower** (6.14 lower to 3.62 lower) | ⨁◯◯◯ Very low^a,b^ | IMPORTANT |
| **CRP** | | | | | | | | | | | | |
| 12 | randomised trials | serious^a^ | very serious^b^ | not serious | not serious | none | 565 | 563 | - | MD **2.26 lower** (2.76 lower to 1.76 lower) | ⨁◯◯◯ Very low^a,b^ | IMPORTANT |
| **NO** | | | | | | | | | | | | |
| 6 | randomised trials | serious^a^ | not serious | not serious | not serious | none | 265 | 260 | - | MD **11.56 higher** (9.37 higher to 13.76 higher) | ⨁⨁⨁◯ Moderate^a^ | IMPORTANT |
| **ET-1** | | | | | | | | | | | | |
| 4 | randomised trials | serious^a^ | serious^b^ | not serious | not serious | none | 208 | 193 | - | MD **16.21 lower** (18.39 lower to 14.02 lower) | ⨁⨁◯◯ Low^a,b^ | IMPORTANT |

**CI:** confidence interval; **MD:** mean difference; **RR:** risk ratio

**Explanations**

a. The allocation and/or blinding were not clear.

b. The included studies exhibited heterogeneity.

# Summary of Adverse Drug Reactions (ADRs) from Included Studies. Note: T: trail group; C: control group.

| Types of Adverse Reactions | Number of incidences  /Overall sample size in T | Number of incidences  /Overall sample size in C | P |
| --- | --- | --- | --- |
| Gastrointestinal adverse reactions | 18/356 | 23/353 | P = 0.4 |
| Bleeding | 9/229 | 17/226 | P = 0.11 |
| Dizziness and headache | 2/141 | 4/141 | P = 0.45 |
| Dry mouth | 1/45 | 1/45 | / |
| fever | 1/45 | 0/45 | / |
| hypotension | 2/50 | 3/50 | / |
| Acute/subacute thrombosis | 0/60 | 2/60 | / |
| thrombocytopenia | 1/60 | 1/60 | / |
