# Supplementary figures and images for: Tongxinluo capsule for acute myocardial infarction: a systematic review and meta-analysis
Source: Front Pharmacol. 2025 Oct 22;16:1632809. doi: 10.3389/fphar.2025.1632809 (PMC12587000; doi:10.3389/fphar.2025.1632809)

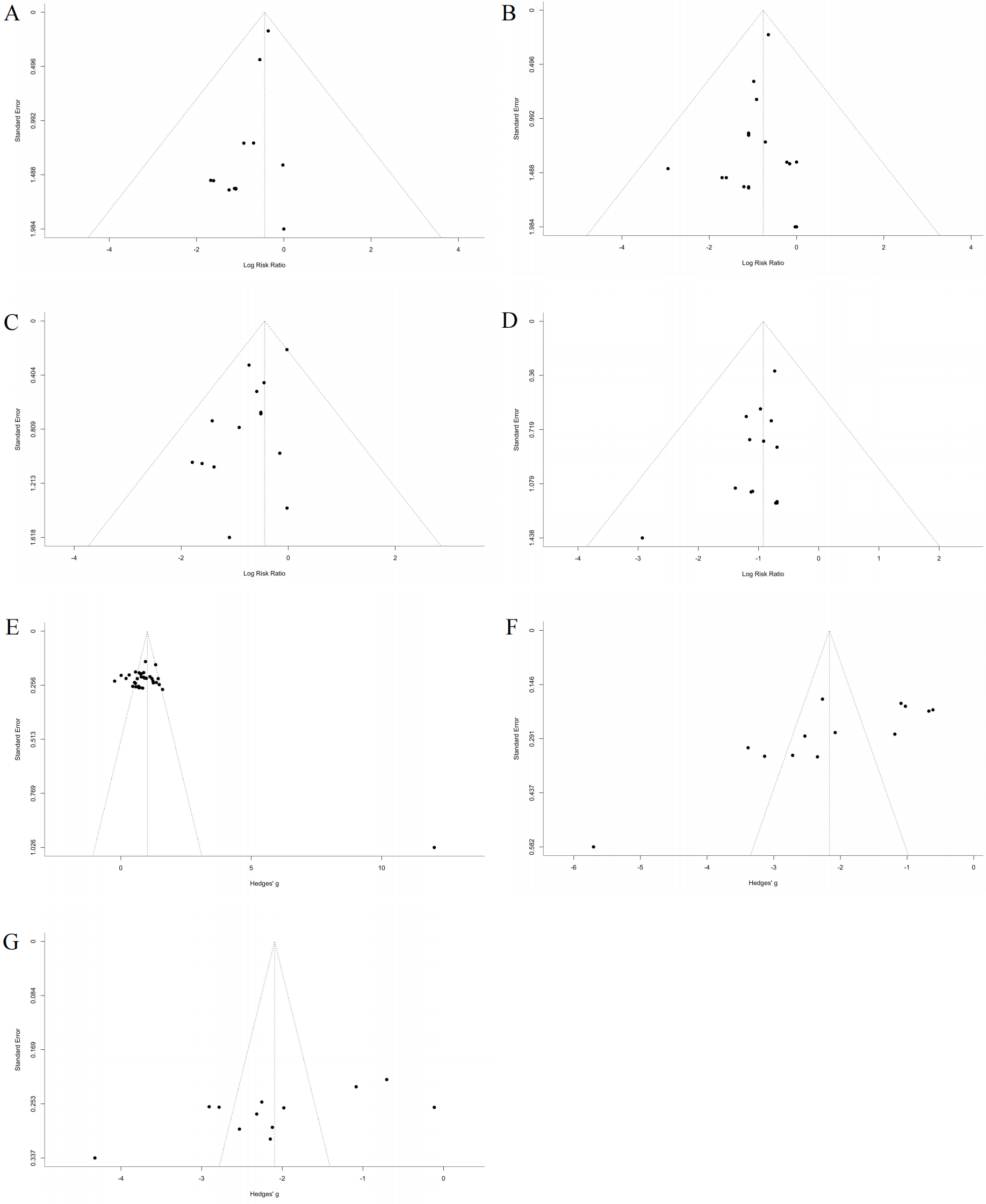

Supplement: Supplementary file 2 [file Image1.tif]
